# Supplementary material for: Prevalence and significance of combined low muscle volume and high muscle fat in patients with Child-Pugh A-B cirrhosis
Source: JHEP Rep. 2026 Sep 11;8(12):102031. doi: 10.1016/j.jhepr.2026.102031 (PMC13742102; doi:10.1016/j.jhepr.2026.102031)

Prevalence and significance of combined low muscle volume and high muscle fat in patients with Child-Pugh A-B cirrhosis

Mikael Fredrik Forsgren, Daniel Sjögren, Henrik Stjernman, Patrik Nasr, Wile Balkhed, Anna Cederborg, Markus Holmberg, Wolf Bartholomä, Cecilia Jönsson, Peter Lundberg, Martin Rejler, Nils Dahlström, Stergios Kechagias, Olof Dahlqvist Leinhard, Mattias Ekstedt

Table of contents

Supplementary tables.. 2

Supplementary figures 6

Supplementary tables

Table S1 Complete list of ranked liver cirrhosis aetiologies in alphabetical order.

**Aetiologies**	**n**
AAT-deficiency	1
AIH	9
AIH-PBC overlap	1
AIH, ALD, MASLD	1
ALD	50
ALD, HBV, HCV	1
ALD, HCV	1
ALD, HFE	1
ALD, MASLD	9
ALD, MASLD, HCV	1
ALD, MASLD, HCV, HBV	1
Budd-Chiari secondary to polycythemia vera	1
Cryptogenic	7
CVID	1
HCV	4
HCV, ALD	5
HCV, ALD, MASLD	2
HFE	1
MASLD	36
MASLD, ALD	5
MASLD, Cryptogenic	1
MASLD, Other	1
Neonatal cholestasis	1
Other	1
PBC	5
PBC, AIH	1
Primary ciliopathy	1
SSC	1

AAT, alpha-1 antitrypsin; AIH, autoimmune hepatitis; ALD, alcohol-related liver disease; CVID, common variable immunodeficiency; HBV, hepatitis-B virus; HCV, hepatitis-C virus; HFE, hereditary hemochromatosis; MASLD, metabolic-dysfunction associated steatotic liver disease; PBC, primary biliary cholangitis; SSC, secondary sclerosing cholangitis.

Table S2 Patient demographics, body composition, and functional test differences between the low and normal muscle volume. Data presented as mean (standard deviation), n (%), or median [range] as applicable. 147 patients had a complete thigh muscle assessment.

	**Low Muscle Volume** n = 80 (54%)	**Normal Muscle Volume** n = 67 (46%)	**P-values**	**Test**
Sex [male]	50 (32%)	35 (52%)	0.210	Chi2
Age [years]	65.8 (8.8)	62.8 (12.3)	0.088	T-test
BMI [kg/m^2]	28.5 (5.5)	30.3 (5.6)	**0.046**	T-test
Disease history				
Diabetes Type 2	32 (37%)	25 (37%)	0.739	Chi2
Primary aetiology ALD	44 (55%)	19 (28%)	**0.001**	Chi2
Primary aetiology MASLD	15 (19%)	27 (40%)	**0.004**	Chi2
Primary aetiology Hepatitis C	6 (8%)	5 (7%)	0.993	Chi2
Primary aetiology AIH or PBC	8 (10%)	8 (12%)	0.707	Chi2
Primary other aetiology	7 (6%)	8 (12%)	0.525	Chi2
Decompensation event prior to baseline	26 (33%)	19 (28%)	0.587	Chi2
Child-Pugh Class B	9 (11%)	6 (9%)	0.691	Chi2
MELD 3.0 [-]	8 [7, 10]	8 [7, 9]	0.885	Mann-Whitney U
MRI-based body composition				
Visceral Adipose Tissue volume [L]	5.8 (2.7)	5.5 (2.6)	0.574	T-test
Abdominal Subcutaneous Adipose Tissue volume [L]	8.9 (3.9)	9.2 (4.6)	0.646	T-test
Total Thigh Fat-free Muscle volume [L]	9.3 (2.2)	11.2 (2.6)	**<.001**	T-test
Mean Anterior Thigh Muscle Fat Infiltration [%]	9.0 (3.0)	6.9 (2.0)	**<.001**	T-test
Liver Proton Density Fat Fraction [%]	3.7 [2.2, 7.3]	3.3 [2.1, 5.6]	0.448	Mann-Whitney U
L3 Skeletal Muscle Index (L3-SMI) [cm^2/m^2]	43.6 (9.5)	50.9 (10.0)	**<.001**	T-test
Muscle Assessment Score (MAsS)				
Muscle Volume Z Score [SDs]	-1.6 (0.9)	0.3 (0.7)	**<.001**	T-test

Mean Anterior Thigh Muscle Fat Infiltration (sex-adjusted) [pp]	1.1 (2.8)	-1.1 (1.8)	**<.001**	T-test
Physical and cognitive function tests				
Animal Naming Test (ANT)	23 [18, 28]	21 [19, 25]	0.696	T-test
Short Physical Performance Battery (SPPB)	10 [9, 12]	11 [9, 12]	0.405	Mann-Whitney U
Liver Frailty Index (LFI)	3.7 [3.3, 4.1]	3.5 [3.1, 4.0]	0.108	Mann-Whitney U
Hand grip strength (HGS)	34 [24, 39]	36 [25, 46]	0.114	Mann-Whitney U
Dichotomous physical and cognitive function outcomes				
Low ANT	7 (9%)	6 (9%)	0.920	Chi2
Sarcopenia by L3-SMI	49 (62%)	14 (21%)	**<0.001**	Chi2
Low to very low physical performance by SPPB	6 (8%)	2 (3%)	0.222	Chi2
Frail by LFI	11 (14%)	7 (11%)	0.530	Chi2
Low hand-grip strength by HGS	11 (14%)	9 (14%)	0.960	Chi2

ALD, alcohol-related liver disease; MASLD, metabolic-dysfunction associated steatotic liver disease; AIH, autoimmune hepatitis; PBC, primary biliary cholangitis; MELD, Model for End-stage Liver Disease; ANT, Animal Naming Test; SPPB, Short Physical Performance Battery; LFI, Liver Frailty Index; HGS, Hand Grip Strength; L3-SMI, Skeletal Muscle Index at the 3rd Lumbar vertebrae.

Table S3 History of decompensation events prior to inclusion against the Child-Pugh score at inclusion. Data presented as n (%), p-value for fraction of historical events in the Child-Pugh A versus Child-Pugh B group using the Chi^2-test.

**Decompensation history** (prior to inclusion)	**Total**	**A5**	**A6**	**B7**	**B8**	**B9**	**P-value** (Child-Pugh A vs. B)
Clinical ascites	39 (26%)	25 (64%)	7 (18%)	5 (13%)	2 (5%)	0	0.054
Acute kidney injury	17 (11%)	12 (71%)	4 (24%)	1 (6%)	0	0	0.548
Variceal bleeding	14 (9%)	6 (43%)	5 (36%)	3 (21%)	0	0	0.134
Encephalopathy	8 (5%)	3 (38%)	3 (38%)	1 (13%)	0	1 (13%)	0.146
TIPS treatment	4 (3%)	1 (25%)	3 (75%)	0	0	0	0.499
Portal vein thrombosis	3 (2%)	2 (67%)	1 (33%)	0	0	0	0.560
Spontaneous bacterial peritonitis	1 (1%)	0	0	0	1 (100%)	0	**0.003**
Varices	69 (46%)	50 (72%)	12 (17%)	6 (9%)	1 (1%)	0	0.956

Supplementary figures

Fig. S1 Correlation analysis of muscle measurements to functional tests.

Spearman rank correlation analysis between thigh Muscle Fat Infiltration (A, D, G), thigh Muscle Volume Z-score (B, E, H), and thigh Fat-Free Muscle Volume (C, F, I), against the L3 Skeletal Muscle Index (A, B, C), L3 Skeletal Muscle Fat Infiltration (D, E, F), and the Liver Frailty Index (G, H, I).

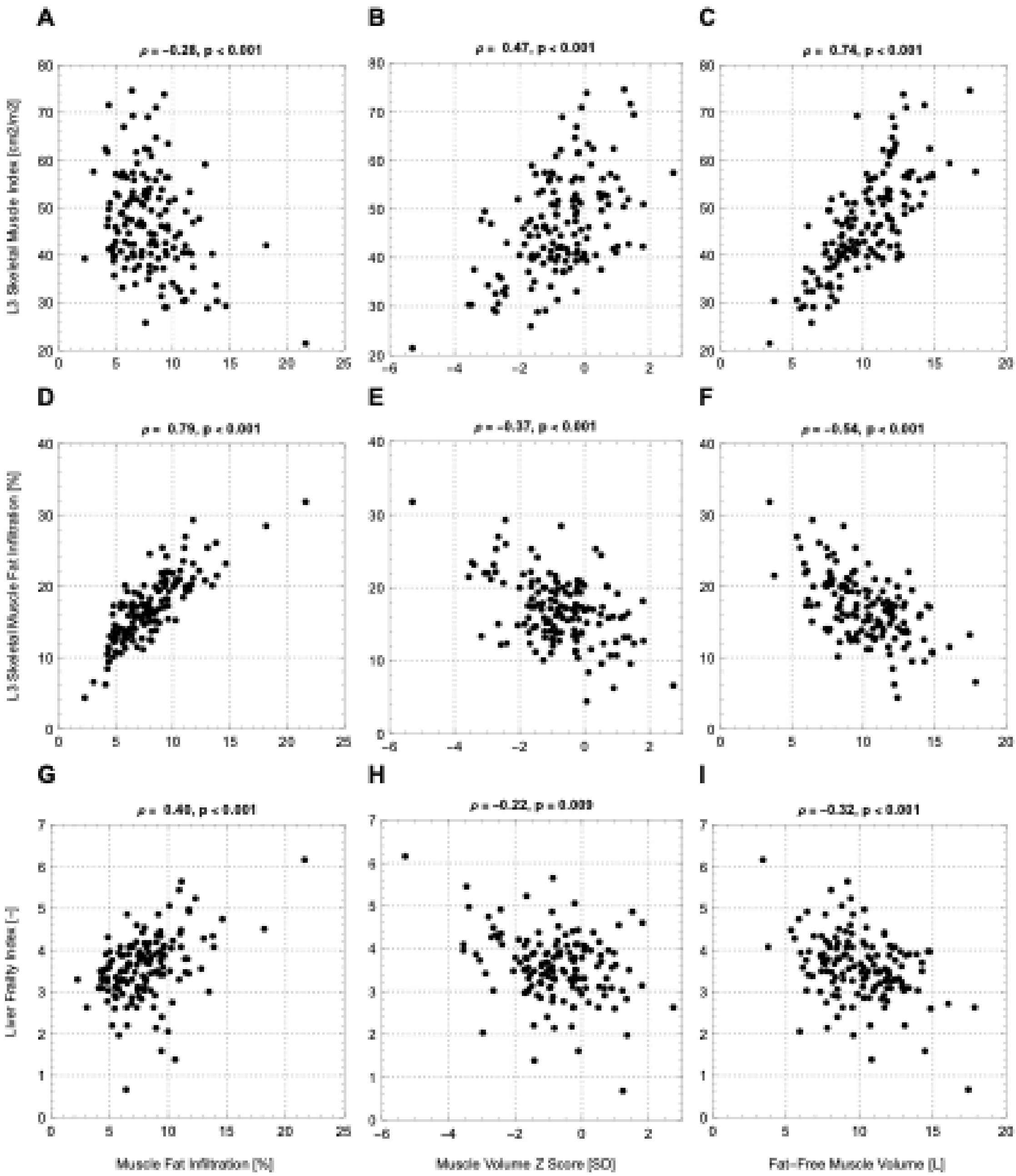

Fig. S2 Correlation analysis of muscle measurements to functional tests.

Spearman rank correlation analysis between thigh Muscle Fat Infiltration (A, D, G), thigh Muscle Volume Z-score (B, E, H), and thigh Fat-Free Muscle Volume (C, F, I), against the Gait Speed (A, B, C), Hand Grip Strength (D, E, F), and the Short Physical Performance Battery (G, H, I).

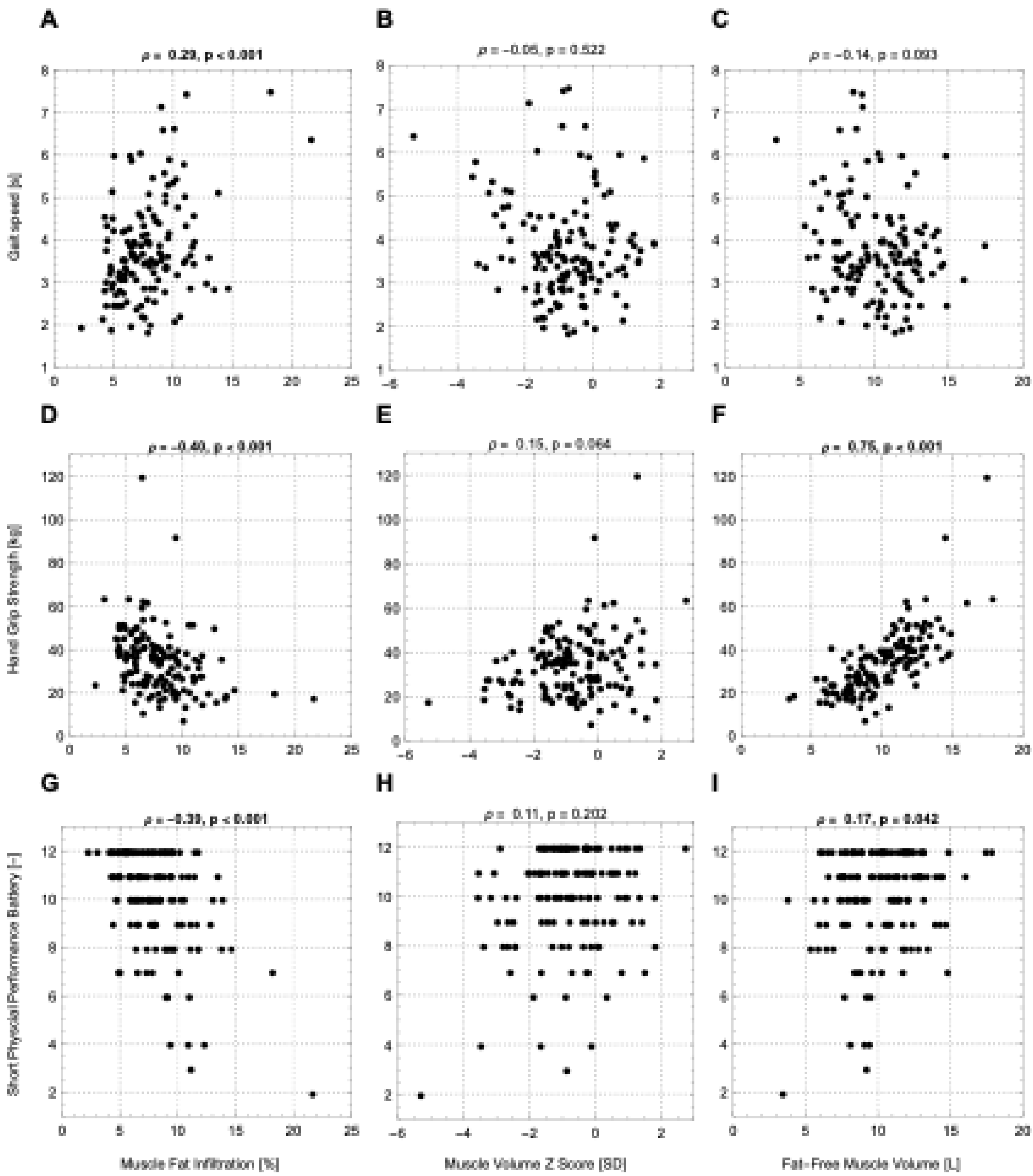

Supplement: Supplementary file 1 [file mmc1.pdf]
