# Supplementary material for: Prevalence and significance of combined low muscle volume and high muscle fat in patients with Child-Pugh A-B cirrhosis
Source: JHEP Rep. 2026 Sep 11;8(12):102031. doi: 10.1016/j.jhepr.2026.102031 (PMC13742102; doi:10.1016/j.jhepr.2026.102031)

Research article

JHEP Reports

Prevalence and significance of combined low muscle volume and high muscle fat in patients with Child-Pugh A-B cirrhosis

Authors

Mikael Fredrik Forsgren, Daniel Sjögren, Henrik Stjernman, …, Stergios Kechagias, Olof Dahlqvist Leinhard, Mattias Ekstedt

Correspondence

mikael.f.forsgren@liu.se (M.F. Forsgren).

Graphical abstract

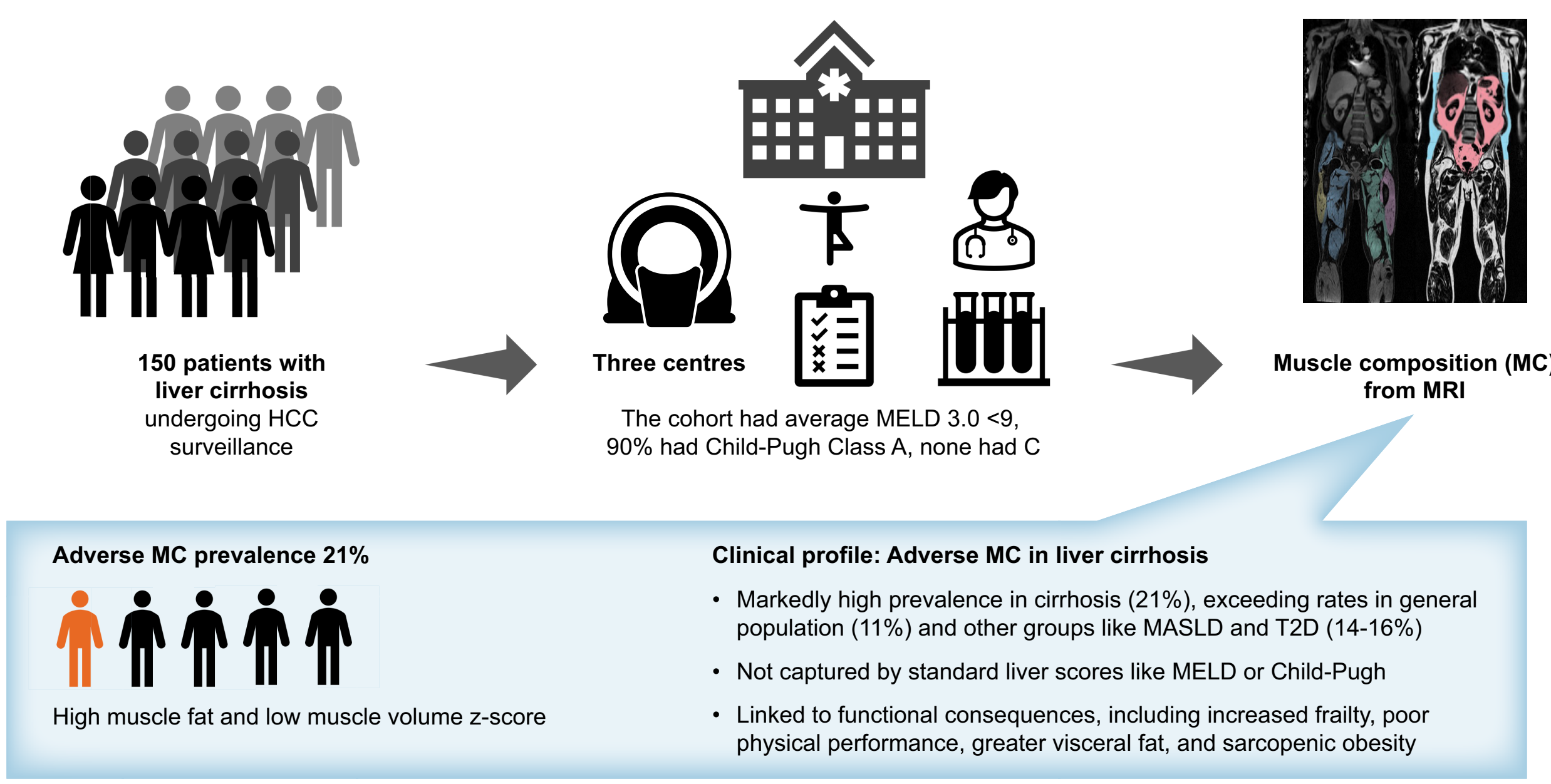

Highlights:

- MRI-based HCC surveillance can, with a short addon, also assess muscle composition.
- Adverse muscle composition (AMC) combines low muscle volume and high muscle fat.
- The AMC phenotype is highly prevalent (21%) in patients with Child-Pugh A-B cirrhosis.
- The AMC phenotype is not captured by standard liver scores.
- Patients with cirrhosis and AMC exhibit higher frailty and impaired physical function.

Impact and implications:

This study provides scientific justification for utilising a concise magnetic resonance imaging (MRI) protocol to assess muscle health during MR-based HCC surveillance in patients with liver cirrhosis, demonstrating its feasibility as a combined MRI-based tool for integrated diagnostics. By effectively identifying patients with adverse muscle composition, linked to higher frailty and poorer physical performance, our findings highlight a patient cohort that warrants future targeted research and the development of specific clinical strategies. The findings suggest that assessing both muscle fat and volume provides a more comprehensive understanding of muscle health than assessing muscle volume alone, potentially leading to more effective management strategies. Future longitudinal data from ACCESS-ESLD will be vital to further explore long-term outcomes and inform the clinical utility of such integrated assessments, ultimately informing refined clinical practices and patient care.

https://doi.org/10.1016/j.jhepr.2026.102031
© 2026 The Authors. Published by Elsevier B.V. on behalf of European Association for the Study of the Liver (EASL). This is an open access article under the CC BY license (http://creativecommons.org/licenses/by/4.0/).

JHEP|Reports

Prevalence and significance of combined low muscle volume and high muscle fat in patients with Child-Pugh A-B cirrhosis

Mikael Fredrik Forsgren[1,2,3,*], Daniel Sjögren[1,4], Henrik Stjernman[5], Patrik Nasr[1,3,6,7], Wile Balkhed[6], Anna Cederborg[1,8,9], Markus Holmberg[1], Wolf Bartholomä[1,3,10], Cecilia Jönsson[1], Peter Lundberg[1,3,10,11], Martin Rejler[12,13], Nils Dahlström[1,3,10], Stergios Kechagias[1,6], Olof Dahlqvist Leinhard[1,2,3], Mattias Ekstedt[1,3,6]

JHEP Reports **2026**. vol. 8 | 1–12

Check for updates

Background & Aims: Physical frailty and sarcopenia are linked to adverse outcomes and higher healthcare utilisation in liver cirrhosis. A magnetic resonance imaging (MRI) assessment combining thigh muscle volume and muscle fat infiltration identifies muscle composition (MC) phenotypes, where adverse MC (high fat, low volume) independently predicts all-cause mortality. We aimed to determine MC prevalence, correlate MRI measurements with functional and physical performance tests, and characterise MC groups in patients with liver cirrhosis undergoing hepatocellular carcinoma (HCC) surveillance.

Methods: The ACCESS-ESLD study prospectively enrolled 150 patients with liver cirrhosis. An abbreviated MRI (for HCC surveillance and an assessment of muscle quantity and body fat), blood sampling (Child-Pugh, model for end-stage liver disease [MELD] 3.0), a Short Physical Performance Battery (SPPB) test, and a Liver Frailty Index (LFI) test were performed.

Results: Out of 150 patients (57% males, mean age 64 ± 11 years, BMI 29.3 ± 5.6 kg/m^2), 44% had alcohol-related liver disease and 28% had metabolic dysfunction-associated steatotic liver disease. Ninety per cent were Child-Pugh A, and the mean MELD was 8.9. Adverse MC was found in 21% of patients, who showed higher LFI, poorer SPPB, and 1.6 L more visceral fat. No differences in Child-Pugh, MELD, decompensation history, type 2 diabetes, or BMI were observed. Muscle measurements strongly correlated with frailty and physical tests, with muscle fat most strongly linked to the LFI and the SPPB.

Conclusions: Incorporating a concise MRI protocol to assess muscle health into HCC surveillance effectively identifies one in five patients with higher frailty and poorer physical performance, independent of liver disease status. Assessing both muscle fat and volume provides additional benefit beyond muscle volume alone.

Clinical Trials Registration: The study is registered at ClinicalTrials.gov (NCT05502198).

© 2026 The Authors. Published by Elsevier B.V. on behalf of European Association for the Study of the Liver (EASL). This is an open access article under the CC BY license (http://creativecommons.org/licenses/by/4.0/).

Introduction

Approximately 1.5 billion individuals worldwide are affected by chronic liver disease.[1] The end stage of all chronic liver diseases is liver cirrhosis, which is linked to an increased risk of all-cause mortality and liver-related events such as decompensation and hepatocellular carcinoma (HCC), leading to ∼2 million deaths worldwide every year.[2] Furthermore, sarcopenia, characterised by the progressive loss of muscle volume and function, is another common and major complication in patients with cirrhosis. Previous research indicates that sarcopenia is present in approximately 38% of patients with cirrhosis and that it is associated with increased morbidity, mortality, and poorer treatment results.[3] Recognising sarcopenia as a prognostic risk factor in cirrhosis, recent clinical guidance recommends systematic assessment of muscle loss to evaluate the risk for poor outcomes in these patients.[3,4] In addition, the clinical manifestation of muscle dysfunction in terms of physical frailty is often assessed with different types of physical strength and function tests. Common tests include hand grip strength (HGS) and the Liver Frailty Index (LFI), which focus on physical frailty, as well as the Short Physical Performance Battery (SPPB), which has a greater emphasis on the physical function of the lower extremities.[4] Cognitive functional impairment may be assessed using the Animal Naming Test (ANT), which is a test for hepatic encephalopathy in patients with liver cirrhosis.[5]

While several methods exist to evaluate body and muscle composition (MC), imaging techniques such as magnetic resonance imaging (MRI) enable us to benefit from images taken as part of standard practice and to complement clinical examinations with concise sequences tailored for quantifying body composition.[6] MRI is considered the gold standard for body composition assessment, and methods for objective measurement of muscle health unbiased by BMI have been developed,[7] which have also been referred to as the Muscle Assessment Score (MAsS). The components of the MAsS, including the thigh muscle volume z-score (MVZ) and muscle fat infiltration (MFI), can be used to define MC phenotypes.[8] Among these, adverse MC (characterised by a low MVZ and

* Corresponding author. Address: Department of Health, Medicine and Caring Sciences, Linköping University, SE-581 83 Linköping, Sweden.
E-mail address: mikael.f.forsgren@liu.se (M.F. Forsgren).
https://doi.org/10.1016/j.jhepr.2026.102031

high MFI) has been shown to be an independent predictor of all-cause mortality and can be used to identify vulnerable patients with steatotic liver disease and in the general population in the UK Biobank.[8–10] MVZ is based on the fat-fat free muscle volume (FFMV) of the thighs, which is a direct measurement of the viable muscle volume in the thigh. The thigh MVZ, however, describes whether the amount of viable muscle volume in the thighs is smaller or larger than expected given the patient's overall body size and sex, and is a marker of sarcopenia.[7] MC biomarkers, such as those described above and based on assessment of muscles on a slice at the third lumbar vertebrae using MRI or computed tomography (CT), have been shown to be linked to poor performance in physical function tests such as LFI and HGS as well as self-reported function.[7,11,12]

The field of liver cirrhosis is shifting from reactive (treating decompensation once it occurs) to proactive (preventing decompensation from happening). For this shift to be successful, validated biomarkers that predict prognosis and reflect changes in disease activity are needed. In addition, current guidelines recommend screening for HCC biannually with ultrasonography (US), CT, or MRI, with US being the most commonly used modality.[13] However, recent studies have reported MRI to provide a higher HCC detection rate compared to US, with abbreviated MRI (aMRI) showing high sensitivity for detecting HCC.[14]

In these analyses of the baseline examination of the ACCESS-ESLD study,[15] we aimed to: (i) determine the prevalence of MC phenotypes in patients undergoing HCC surveillance with aMRI; (ii) correlate MRI-based muscle measurements with physical and cognitive function as well as frailty tests; and (iii) characterise the functional performance and demographics of MC phenotypes.

Patients and methods

Study design and patients

In the ACCESS-ESLD study we prospectively recruited 150 adults with liver cirrhosis undergoing HCC surveillance at the Gastroenterology and Hepatology clinics at the University Hospital in Linköping, the District Hospital in Eksjö, and the County Hospital in Jönköping.[15] Patients were included based on the Swedish national guidelines for HCC surveillance, which recommend surveillance for patients with Child-Pugh A-B cirrhosis, acute porphyria (≥50 years), and high-risk non-cirrhotic hepatitis B. Surveillance is explicitly excluded for Child-Pugh C patients not eligible for transplant and non-cirrhotic F3 fibrosis cases caused by HCV or metabolic dysfunction-associated steatotic liver disease (MASLD).[16] Of the 150 patients recruited, 100 were enrolled at the University Hospital of Linköping (which serves as both a primary and secondary care facility) and 50 at the primary clinics in Eksjö and Jönköping. Patients were recruited between 20 May 2021, and 7 March 2024. Informed consent was obtained from all patients in accordance with the Declaration of Helsinki (2018). The ACCESS-ESLD study was approved by the Swedish Ethical Review Authority (Dnr. 2020-07215, with amendments Dnr. 2022-02902-02 and 2022-06142-02) and registered as a clinical trial (NCT 05502198). Although follow-up data were obtained, this paper only assessed cross-sectional baseline visit data.

The primary aetiology of cirrhosis was determined from patient records. Because of the lack of reliable data on alcohol consumption at the time of diagnosis, it was not possible to accurately apply the 'MASLD and increased alcohol intake' (MetALD) subclassification.[17] Therefore, for this analysis, only MASLD and alcohol-related liver disease (ALD) were used as distinct primary aetiologies.

Clinical variables

Blood, serum, and plasma workups, along with the medical history of patients in ACCESS-ESLD, have been described elsewhere.[15] This paper includes data on basic patient information, diabetes, cirrhosis history, blood tests (platelets, prothrombin time, albumin, bilirubin), and common liver disease severity scores (the Child-Pugh score and the model for end-stage liver disease [MELD] score) which were acquired within a week of imaging, usually on the same day.

Magnetic resonance imaging

The entire 20 min MRI examination is described in the published study protocol.[15] The present study utilised the muscle and fat distribution assessment, which was based on a rapid, 8–10 min, non-contrast dual-echo Dixon MRI protocol, providing water- and fat-separated volumetric data from neck to knee, as well as a six-echo Dixon MRI protocol providing volumetric data for liver fat assessment. The body composition analysis included thigh FFMV, MFI, visceral adipose tissue (VAT), abdominal subcutaneous adipose tissue (ASAT) volumes, the skeletal muscle area as well as the muscle fat infiltration at the third lumbar vertebra (L3-SMI and L3-MFI), and liver proton density fat-fraction (PDFF) using AMRA® Researcher (AMRA Medical AB, Linköping, Sweden). The methodology and MR protocol have been published extensively.[15,18,19] Briefly, the analysis involves: (1) automatic image calibration, (2) automatic labelling and registration of fat and muscle regions, (3) quality control (QC) of anatomical regions and MR data by trained personnel at AMRA Medical AB, and (4) quantification of fat and muscle volumes based on the calibrated images. These measurements are comparable across commonly used clinical MRI scanners and field strengths.[20,21] Example images used in the body composition analysis are shown in Fig. 1.

For each patient, the MAsS was calculated, consisting of a sex- and BMI-invariant MVZ and MFI adjusted for sex differences using the median MFI in a sex-specific reference population.[7]. Adverse MC was defined as high MFI (the sex-specific >75th percentile within the UK Biobank) and low MVZ (the sex-specific <25th percentile within the UK Biobank).[7] High MFI, low MVZ, and normal MC phenotypes were also defined using the same cut-off points.[8] In addition, the L3-SMI was calculated by normalising the skeletal muscle area at the third lumbar vertebra with height^2. The cut-off points for defining a low L3-SMI were <50 cm^2/m^2 for males and <39 cm^2/m^2 for females.[22] Sarcopenic obesity was defined in two ways: one based on low MVZ, and one based on low L3-SMI, both with concurrent obesity (BMI ≥30 kg/m^2).

The MC phenotypes, including adverse MC, were defined using thresholds derived from the UK Biobank reference population.[7] While the UK Biobank cohort represents a general community-dwelling population rather than a specific liver cirrhosis cohort, both populations share similar age and ethnic profiles. In this study, all patients were recruited in a compensated state and were community-dwelling at the time of inclusion, supporting the relevance of these reference ranges. Importantly, since our cohort consisted of community-dwelling patients with compensated cirrhosis, the extreme fluid shifts

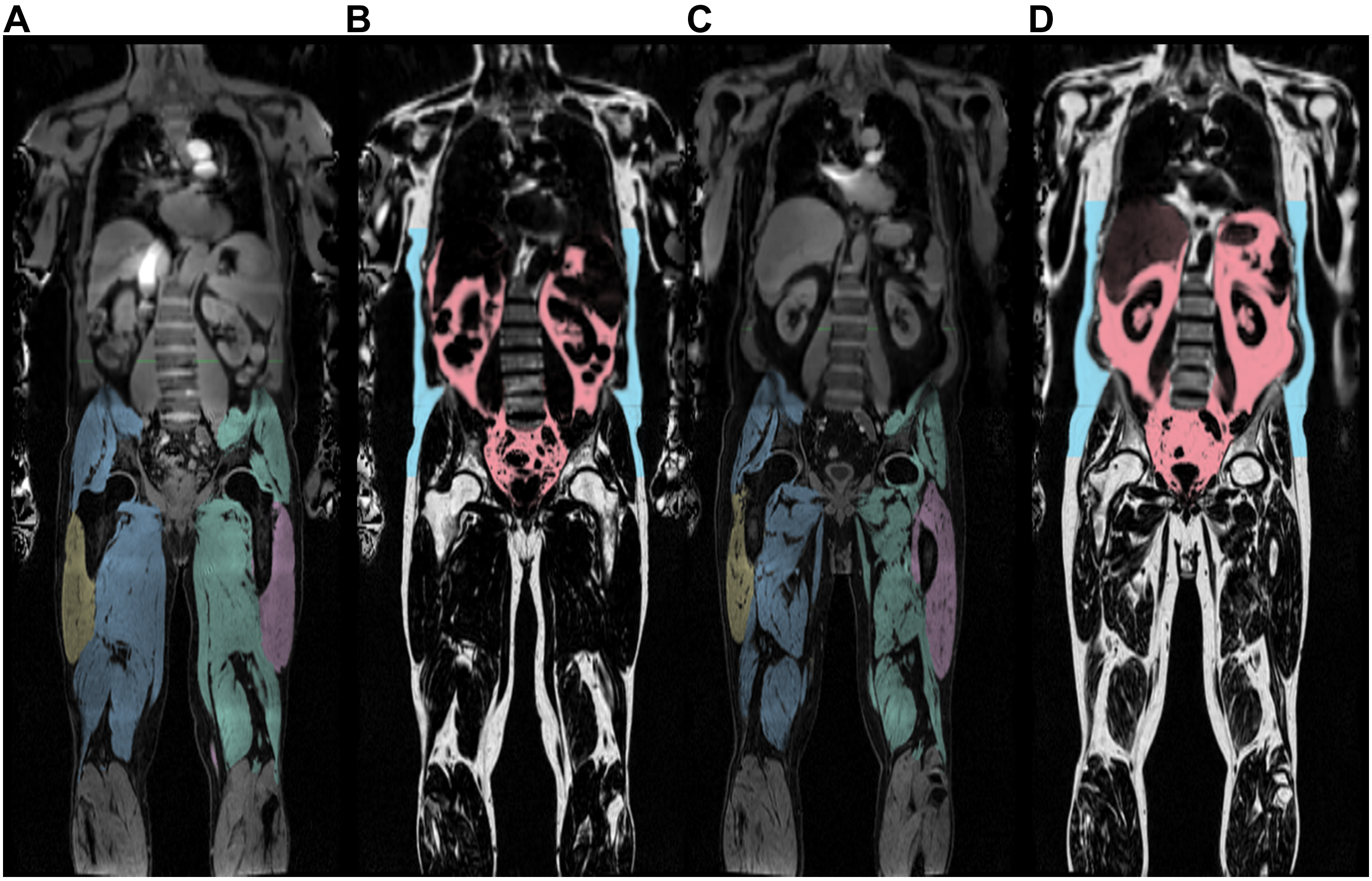

Fig. 1. Fat-water separated MR images with segmentations. The image pairs show two males with similar BMI, 26.7 kg/m^2 and 27.7 kg/m^2, and different ages, 55 years and 75 years, left and right, respectively. The 75-year-old patient (A,B) had normal muscle composition, whereas the 55-year-old patient (C,D) had adverse muscle composition, meaning that there was a high amount of fat in the muscles and a low muscle volume. Panels (B) and (D) show the fat compartments (visceral and subcutaneous adipose tissue volume in red and blue, respectively), and panels (A) and (C) show the water channel with the thigh muscles in false colour.

seen in end-stage disease were less prevalent, making the UK Biobank reference ranges a viable starting point.

The applicability of these population-based cut-offs to chronic liver disease has been previously supported by pilot studies in patients with decompensated cirrhosis and post-liver transplantation.[11,23,24] Furthermore, the primary objective of this study was to evaluate whether general population thresholds retain descriptive value within a cirrhosis cohort, and to describe the prevalence of those MC phenotypes in a liver cirrhosis cohort. To validate these thresholds in a liver cirrhosis cohort, we will evaluate these cut-points against clinical outcomes, including liver-related mortality and decompensation events, which will allow for a direct comparison between UK Biobank-derived and cohort-specific percentiles.

Functional and performance tests

The SPPB, HGS, LFI, and ANT were performed as previously described.[15] Briefly, the SPPB consists of three components: a 4-m gait speed test, a timed chair stand, and balance testing in three positions. The LFI has some overlap with the SPPB and consists of HGS, chair stands, and balance testing. HGS was also assessed independently. The SPPB focuses on lower extremity physical function, the LFI and HGS focus on physical frailty, and the ANT is a cognitive function test developed to assess hepatic encephalopathy. These tests were performed within 1 week of imaging, and in most cases on the same day. Low HGS was defined according to the European Working Group on Sarcopenia in Older People (EWGSOP2) as <27 kg for males and <16 kg for females.[25] Frailty was defined by the LFI as LFI >4.3.[26] With the SPPB, low-to-very-low physical performance was defined as SPPB <7.[27] Low ANT, suggestive of impaired cognitive function (hepatic encephalopathy), was defined as ANT <15 animals.[28]

Statistical analysis

Mathematica version 12.2.0 (Wolfram Research Inc, Champaign, IL, USA) was used for the statistical analysis. A Spearman rank test was used to assess the correlation between the MAsS measurements and muscle functional tests. The X^2 test, Mann-Whitney *U* test, and Student's *t* test were used to assess MC group differences against functional tests, muscle and fat distribution, and basic patient descriptions. To correct for significant MC group differences in disease history and demographics, linear and logistic modelling was used. Adjusted model results are presented as effect size estimates or odds ratios, as applicable, with 95% CIs. A linear regression model was used to evaluate the association between L3-level MFI and anterior thigh MFI. A p value <0.05 was considered statistically significant. Percentages are rounded to the nearest integer. Consequently, totals may not sum exactly to 100%.

Data integrity and bias mitigation

To address potential conflicts of interest and ensure image analytical independence, several measures were implemented. Image analysis was conducted by AMRA Medical AB in a controlled environment compliant with ICH-GCP and FDA 21 CFR Part 11, utilising full audit trails. To mitigate interpretative bias, the AMRA QC staff performed analyses using a double-blinded approach: reviewers were blinded to patient identity, clinical status, functional test results, and time point (baseline *vs.* follow-up). Furthermore, a second AMRA QC staff member performed an independent QC review for every dataset, blinded in the same way as the first QC reviewer.

Following the blinded image analysis, the resulting dataset was transferred to Linköping University (Linköping, Sweden), which served as the governor of the study data. The primary statistical analysis was performed by an investigator (MFF) employed by Linköping University, who did not partake in the image acquisition and processing. The principal investigator (ME) acted as the independent guarantor of the study. All study investigators have access to the complete study data and can conduct independent analysis.

Results

Baseline clinical and demographic characteristics

The study cohort comprised 150 patients with cirrhosis (57% male). The mean BMI was 29.2 kg/m^2, with two-thirds of the cohort being either overweight (25%) or obese (41%). The predominant aetiologies for liver cirrhosis were ALD (43%) and MASLD (28%).

Clinically, the cohort had compensated disease. Ninety per cent of patients were Child-Pugh Class A, and the rest were Class B, the mean MELD 3.0 score was 8.9, and 31% had a history of a prior decompensation event. The median time since diagnosis of cirrhosis was 2.9 years. (See Table 1 for full details and Table S1 for all aetiologies.)

Table 1. Basic description and medical history of the 150 recruited patients.

**Patient characteristics**	
No. of patients	150
Sex (male)	86 (57%)
Age (years)	64.3 (10.6)
BMI (kg/m^2)	29.2 (5.6)
Overweight (25–30 kg/m^2)	38 (25%)
Obese (≥30 kg/m^2)	61 (41%)
**Diabetes**	
Diabetes Type 1	1 (1%)
Diabetes Type 2	59 (39%)
Diabetes managed by diet	24 (16%)
Diabetes managed by medicines	50 (33%)
Diabetes managed by insulin	27 (18%)
**Liver disease history**	
Time from cirrhosis diagnosis to baseline visit (years)	2.9 [0.0; 33.4]
Primary aetiology	
ALD	64 (43%)
MASLD	43 (29%)
Hepatitis C	11 (7%)
Autoimmune hepatitis*	11 (7%)
Cryptogenic	7 (5%)
Primary biliary cholangitis	6 (4%)
Hemochromatosis	1 (1%)
AAT deficiency	1 (1%)
Other	6 (4%)
Decompensation event before baseline	47 (31%)
History of clinical ascites	39 (26%)
History of acute kidney injury	17 (11%)
History of variceal bleeding	14 (9%)
History of encephalopathy	8 (5%)
History of TIPS treatment	4 (3%)
History of portal vein thrombosis	3 (2%)
History of spontaneous bacterial peritonitis	1 (1%)
History of varices	69 (46%)
**Blood tests**	
Thrombocytes (×10^{-9}/L)	156 (65)
PK (INR) (-)	1.1 (0.2)
Albumin (g/L)	38.9 (4.9)
Bilirubin (μmol/L)	15.4 (10.2)
Child-Pugh (-)	5.4 (0.8)
A5	114 (76%)
A6	21 (14%)
B7	11 (7%)
B8	2 (1%)
B9	2 (1%)
MELD (-)	8.3 (2.7)
MELD-Na (-)	8.6 (2.8)
MELD 3.0 (-)	8.9 (2.6)

Data are presented as mean (± one standard deviation), n (%), or median [range] as applicable. *Includes one AIH-PBC overlap. AAT, alpha-1 antitrypsin; AIH-PBC, autoimmune hepatitis-primary biliary cholangitis; ALD, alcohol-related liver disease; MASLD, metabolic dysfunction-associated steatotic liver disease; MELD, model for end-stage liver disease; TIPS, transjugular intrahepatic portal shunt.

Data completeness

Of the 150 patients enrolled in the study, 147 (98%) provided complete and usable MRI thigh assessments. Data were excluded for three patients because of claustrophobia (n = 1), motion artefacts (n = 1), or metallic implants (n = 1). Similarly, the functional and cognitive tests were completed by nearly all participants, with data available for 142 to 146 patients across all measures (SPPB: n = 146; ANT: n = 145; HGS: n = 145; LFI: n = 142).

Muscle composition phenotypes

We classified patients into four distinct MC phenotypes (Fig. 2). Forty per cent (40%) of the cohort had a normal MC. Of the 60% with an abnormal phenotype, 33% had isolated low MVZ, 5% had isolated high MFI, and 21% had a combination of both low MVZ and high MFI, defined as adverse MC.

We then stratified by Child-Pugh class and found that the prevalence of MC phenotypes increased with disease severity. Child-Pugh Class A (n = 132): Most patients (41%) had normal MC. Of those with abnormalities, 35% had isolated low MVZ, 5% had isolated high MFI, and the combined adverse MC phenotype was present in 19%. Child-Pugh Class B (n = 15): Only 33% had normal MC. In this group, the prevalence of abnormalities was higher; 20% had isolated low MVZ, 7% had isolated high MFI, and the adverse MC phenotype was twice as prevalent at 40%.

Muscle quality, not just quantity, correlates with functional impairment

To understand the drivers of functional status, we correlated MRI-based muscle metrics with the performance on functional tests (Figs S1 and S2).

Higher MFI, indicating poorer muscle quality, was significantly correlated with enhanced frailty (LFI: $\rho = 0.40$, $p < 0.001$) and worse performance on functional tests (HGS: $\rho = -0.40$, $p < 0.001$; SPPB: ($\rho = -0.39$, $p < 0.001$). It was also correlated with

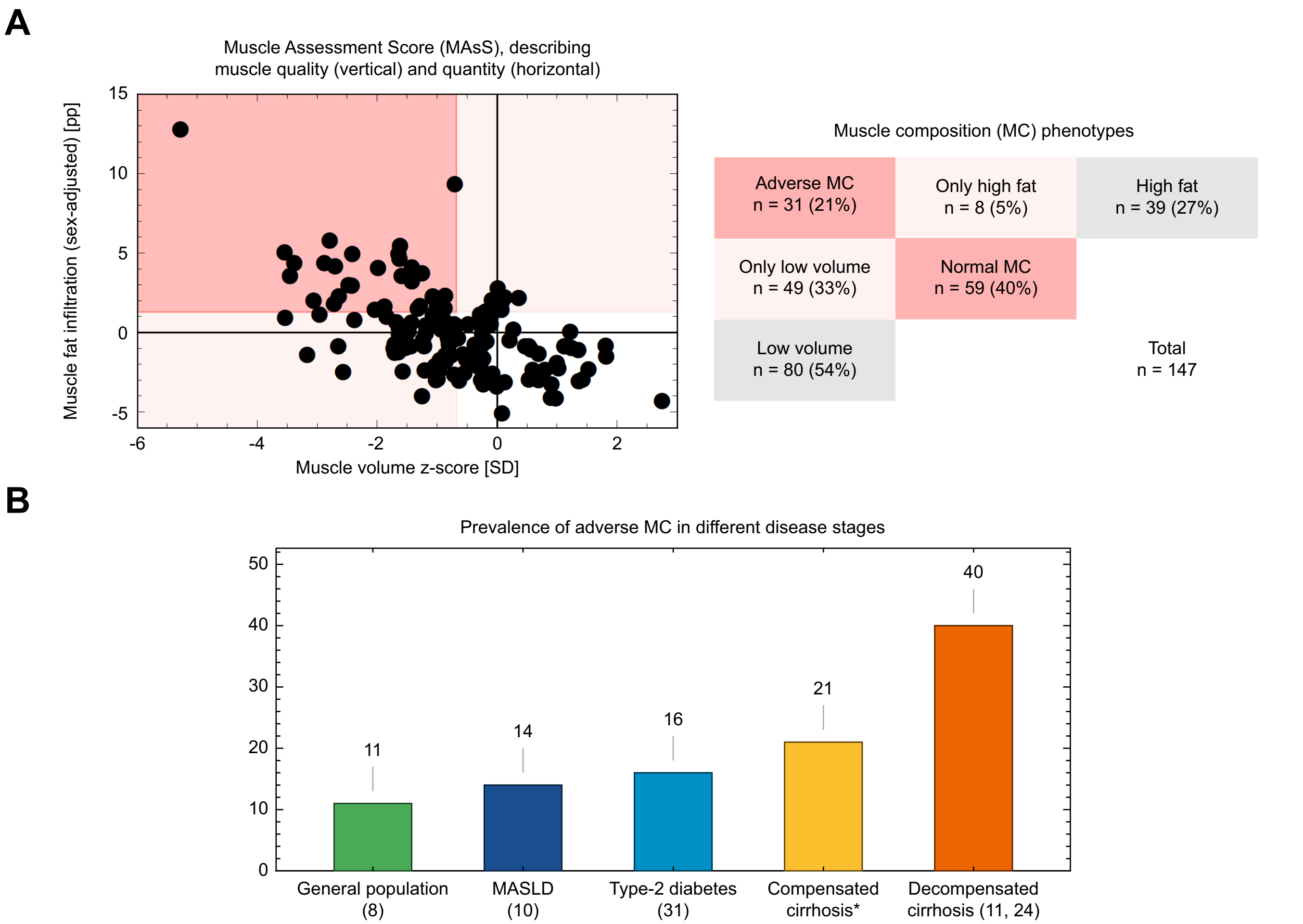

Fig. 2. Muscle composition (MC) phenotype prevalences. (A) Muscle Assessment Score (MAsS) for the patients as well as indications for MC phenotypes. The y-axis shows the muscle fat indicating the quality of the muscles. The x-axis shows the muscle quantity as a z-score – a negative number indicates smaller muscles than expected given the patient's sex and BMI. The MAsS-based MC phenotypes are shown with a red/pink/white background according to the legend on the right. (B) Prevalences of adverse MC across various disease stages, including the present cohort (*). The number in brackets refers to the references; compensated cirrhosis is the average of two references.

the L3-SMI (ρ = -0.28, p <0.001) and gait speed (ρ = 0.29, p <0.001) and strongly with the L3-MFI (ρ = 0.79, p <0.001). The linear model showed that there was a 6.6 pp intercept and a slope of 1.3 pp (all p <0.001), that is, for every unit increase in thigh MFI there was a 1.3 pp increase in L3-MFI.

Higher MVZ was weakly correlated with lower frailty (LFI: ρ = -0.22, p = 0.009) but had no significant correlation with HGS (ρ = 0.15, p = 0.064) or SPPB (ρ = 0.10, p = 0.233). It was correlated with the L3-SMI (ρ = 0.47, p <0.001) and the L3-MFI (ρ = -0.37, p <0.001).

Higher FFMV correlated significantly with better outcomes on all functional tests (LFI: ρ = -0.32, p <0.001; HGS: ρ = 0.75, p <0.001; SPPB: ρ = 0.17, p = 0.042) and showed a strong correlation with the L3-SMI (ρ = 0.74, p <0.001) and a moderate correlation with the L3-MFI (ρ = -0.54, p <0.001).

Adverse muscle composition is associated with impaired physical function

First, we compared baseline characteristics between patients with adverse (n = 31) and non-adverse (n = 116) MC phenotypes. While the groups were similar in sex, BMI, type 2 diabetes T2D, prior decompensation, MELD score, and prevalence of Child-Pugh Class B (see Table 2), patients with adverse MC were significantly older (mean 68.5 *vs.* 63.3 years, p = 0.002) and more likely to have ALD (65% *vs.* 37%, p = 0.006). The adverse MC phenotype was also associated with 1.6 L greater VAT (p = 0.004), a 2.3% increase in liver fat (p = 0.006), but no difference in ASAT (p = 0.473) (Table 2).

The adverse MC phenotype was strongly associated with poorer physical and cognitive function. On a continuous scale, patients with adverse MC scored significantly lower on the SPPB (-2 points, p <0.001), ANT (-2.5 animals, p = 0.033), and LFI (+0.6 points, p <0.001) (Fig. 3). When applying clinical cut-offs, a significantly higher proportion of the adverse MC group was classified as sarcopenic by the L3-SMI (68% *vs.* 37%, p = 0.002), having low physical performance according to the SPPB (19% *vs.* 3%, p = 0.003), and being frail as assessed with the LFI (33% *vs.* 7%, p <0.001; Fig. 4).

Sarcopenic obesity was more prevalent in the adverse MC group (as determined by either L3-SMI 29% *vs.* 6% [p <0.001]

Table 2. Patient demographics, body composition, and functional test differences between the adverse and non-adverse muscle composition.

	Entire cohort n = 147	Adverse muscle composition n = 31 (21%)	Non-adverse muscle composition n = 116 (79%)	*p* value (group test)	Model corrected for age (effect estimate)	Model corrected for age (*p* value)	Model corrected for age and ALD	Model corrected for age and ALD (*p* value)
Sex (male)	85 (58%)	19 (61%)	66 (57%)	0.660 *	n.a.	n.a.	n.a.	n.a.
Age (years)	64.4 (10.6)	68.5 (7.2)	63.3 (11.1)	**0.002**†	n.a.	n.a.	n.a.	n.a.
BMI (kg/m^2)	29.3 (5.6)	29.8 (6.0)	29.2 (5.5)	0.604†	n.a.	n.a.	n.a.	n.a.
Disease history								
Type 2 diabetes	57 (39%)	13 (45%)	44 (38%)	0.684*	n.a.	n.a.	n.a.	n.a.
Primary aetiology ALD	63 (43%)	20 (65%)	43 (37%)	**0.006***	n.a.	n.a.	n.a.	n.a.
Primary aetiology MASLD	42 (29%)	6 (19%)	36 (31%)	0.201*	n.a.	n.a.	n.a.	n.a.
Primary aetiology Hepatitis C	11 (7%)	1 (3%)	10 (9%)	0.311*	n.a.	n.a.	n.a.	n.a.
Primary aetiology AIH or PBC	16 (11%)	2 (6%)	14 (12%)	0.372*	n.a.	n.a.	n.a.	n.a.
Primary other aetiology	15 (10%)	2 (6%)	13 (11%)	0.437*	n.a.	n.a.	n.a.	n.a.
Decompensation event prior to baseline	45 (31%)	10 (32%)	35 (30%)	0.845*	n.a.	n.a.	n.a.	n.a.
Child-Pugh Class B	15 (10%)	6 (19%)	9 (8%)	0.063*	n.a.	n.a.	n.a.	n.a.
MELD 3.0	8 [7, 9]	8 [7, 11]	8 [7, 9]	0.358‡	n.a.	n.a.	n.a.	n.a.
MRI-based body composition								
Visceral adipose tissue volume (L)	5.6 (2.7)	6.9 (2.5)	5.3 (2.6)	**0.004**†	1.5 (0.5; 2.6)	**0.005**§	1.8 (0.8; 2.9)	**<0.001**§
Abdominal subcutaneous adipose tissue volume (L)	9.0 (4.2)	9.5 (3.5)	8.9 (4.4)	0.473†	n.a.	0.419§	n.a.	0.197§
Total thigh fat-free muscle volume (L)	10.1 (2.6)	9.0 (2.7)	10.5 (2.5)	**0.004**†	-1.1 (-2.1; -0.1)	**0.028**§	n.a.	0.051§
Mean anterior thigh MFI (%)	8.0 (2.8)	11.7 (2.7)	7.0 (1.8)	**<0.001**†	4.4 (3.6; 5.2)	**<0.001**§	4.4 (3.6; 5.2)	**<0.001**§
Liver proton density fat fraction (%)	3.5 [2.2, 6.6]	5.5 [2.5, 9.2]	3.2 [2.1, 5.3]	**0.006**‡	2.8 (1.0; 4.6)	**0.003**§	3.3 (1.4; 5.1)	**<0.001**§
L3 Skeletal Muscle Index (L3-SMI) (cm^2/m^2)	46.9 (10.4)	42.1 (9.8)	48.3 (10.2)	**0.003**†	-4.9 (-8.9; -0.9)	**0.018**§	-4.2 (-8.4; -0.1)	**0.045**§
L3 Muscle Fat Infiltration (L3-MFI) (%)	17.2 (4.5)	22.0 (3.8)	15.9 (3.8)	**<0.001**†	5.2 (3.8; 6.6)	**<0.001**§	5.1 (3.7; 6.6)	**<0.001**§
Muscle assessment score (MAsS)								
Muscle volume z-score (SDs)	-0.8 (1.3)	-2.1 (1.0)	-0.4 (1.1)	**<0.001**†	-1.6 (-2.0; -1.1)	**<0.001**§	-1.5 (-2.0; -1.1)	**<0.001**§
Mean anterior thigh MFI (sex-adjusted) (pp)	0.1 (2.6)	3.8 (2.4)	-0.9 (1.6)	**<0.001**†	4.5 (3.8; 5.2)	**<0.001**§	4.6 (3.8; 5.3)	**<0.001**§
Physical and cognitive function tests								
Animal naming test (ANT) (no. animals)	22 [22, 27]	20 [16, 24]	22.5 [19, 28]	**0.033**†	n.a.	0.090§	n.a.	0.080§
Short physical performance battery (SPPB) (-)	11 [9, 12]	9 [8, 11]	11 [10, 12]	**<0.001**‡	-1.4 (-2.2; -0.7)	**<0.001**§	-1.6 (-2.4; -0.8)	**<0.001**§
Liver frailty index (LFI) (-)	3.6 [3.2, 4.1]	4.1 [3.5, 4.5]	3.5 [3.1, 3.9]	**<0.001**‡	0.5 (0.2; 0.8)	**0.001**§	0.5 (0.2; 0.8)	**<0.001**§
Hand grip strength (HGS) (kg)	35 [24, 42]	32 [20, 38]	36 [25, 42]	0.117‡	n.a.	0.408§	n.a.	0.448§
Gait speed (s)	3.6 [3.1, 4.4]	3.9 [3.5, 4.8]	3.5 [3, 4.2]	**0.024**‡	-0.5 (-0.0; -0.9)	**0.040**§	-0.5 (-0.1; -1.0)	**0.028**§
Dichotomous physical and cognitive function outcomes								
Low ANT	13 (9%)	4 (13%)	9 (8%)	0.387*	n.a.	0.652¶	n.a.	0.588¶
Low muscle volume z-score	80 (54%)	31 (100%)	49 (42%)	**<0.001***	n.a.	n.a.	n.a.	n.a.
Sarcopenia by L3-SMI	63 (43%)	21 (68%)	42 (37%)	**0.002***	3.2 (1.4; 7.6)	**0.008**¶	2.6 (1.1; 6.3)	**0.037**¶
Low-to-very-low physical performance by SPPB	8 (5%)	5 (19%)	3 (3%)	**0.003***	6.1 (1.3; 28.3)	**0.020**¶	5.6 (1.2; 26.5)	0.031¶
Frailty by LFI	18 (13%)	10 (33%)	8 (7%)	**<0.001***	5.6 (1.9; 16.1)	**<0.001**¶	7.3 (2.3 23.4)	**<0.001**¶
Low handgrip strength by HGS	20 (14%)	6 (19%)	14 (12%)	0.311*	n.a.	0.528¶	n.a.	0.599¶
Sarcopenic obesity by low muscle volume z-score and BMI ≥30 kg/m^2	32 (22%)	17 (55%)	15 (13%)	**<0.001***	8.4 (3.3; 21.3)	**<0.001**¶	7.8 (3.0; 20.2)	**<0.001**¶
Sarcopenic obesity by sarcopenia by L3-SMI and BMI ≥30 kg/m^2	16 (11%)	9 (29%)	7 (6%)	**<0.001***	5.5 (1.8; 16.6)	**0.003**¶	4.4 (1.4; 14.1)	**0.012**¶

Data are presented as mean (standard deviation), n (%), or median [IQR] as applicable. Model effects estimates are regression coeffects for linear and odds ratio for dichotomous response variables, respectively, with 95% CIs. Bold *p* values indicate statistical significance (*p* <0.005). A total of 147 patients had a complete thigh muscle assessment. *X^2 test, †*t* test, ‡Mann-Whitney *U* test, §linear models corrected for age as well as age and ALD, ¶logistic models corrected for age as well as age and ALD. ALD, alcohol-related liver disease; MASLD, metabolic dysfunction-associated steatotic liver disease; AIH, autoimmune hepatitis; PBC, primary biliary cholangitis; MELD, model for end-stage liver disease; ANT, Animal Naming Test; SPPB, Short Physical Performance Battery; LFI, Liver Frailty Index; HGS, hand grip strength; L3-SMI, Skeletal Muscle Index at the third lumbar vertebra.

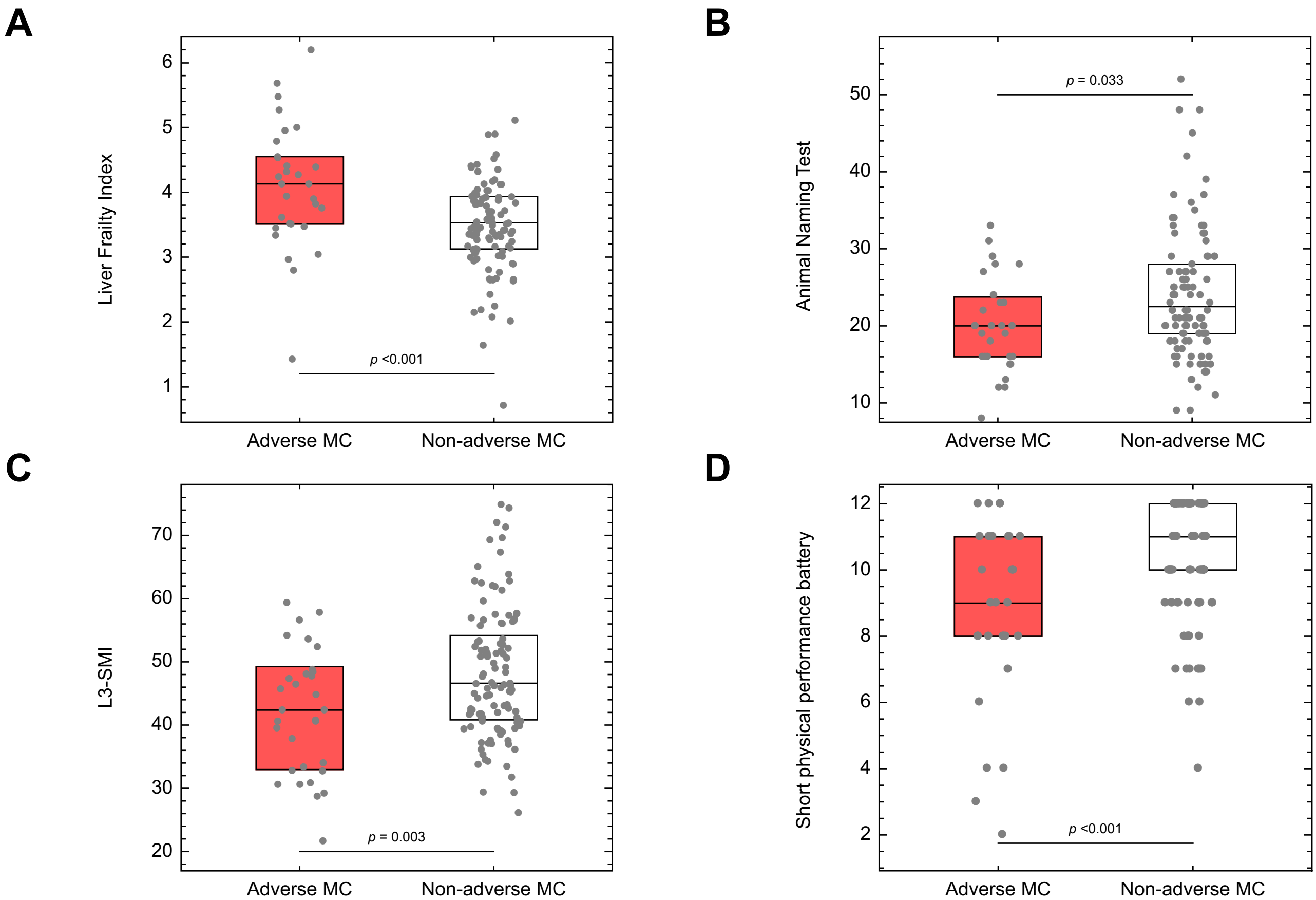

Fig. 3. Adverse muscle composition (MC) and non-adverse MC phenotypes *vs.* functional tests. (A) Liver Frailty Index (Mann-Whitney *U* test), (B) Animal Naming Test (Student's *t* test), (C) L3-SMI (Student's *t* test), and (C) Short Physical Performance Battery (Mann-Whitney *U* test). The box indicates median (line) and IQRs (edges). L3-SMI, Skeletal Muscle Index at the third lumbar vertebra; MVZ, Muscle Volume z-score.

or MVZ 55% *vs.* 13% [*p* <0.001]). This elevated prevalence remained after adjusting for age and ALD aetiology (Table 2 and Fig. 4E,F).

Finally, we performed adjusted analyses correcting for significant baseline differences in disease history and demographics. These associations with impaired physical function (SPPB and LFI) remained significant after adjusting for confounders including age and ALD aetiology (Table 2). In the age-adjusted models, patients in the adverse MC groups had 6.1-fold higher odds for low physical performance, 5.6-fold higher odds for frailty, and 8.4-fold and 5.5-fold higher odds for sarcopenic obesity (as determined by either L3-SMI or MVZ).

To determine whether these functional impairments were driven by low muscle volume alone or by overall adverse MC (*i.e.* high MFI and low MVZ), we conducted a sensitivity analysis. The patients with a low MVZ had significantly lower BMI (*p* <0.046), and a higher proportion had ALD as the primary aetiology (55% *vs.* 28%, *p* = 0.001). We found that patients with a low MVZ did not differ from those with normal muscle volume on any functional or cognitive measure (SPPB, LFI, HGS, or ANT; all *p* >0.05). Patients with a low MVZ had a significantly lower L3-SMI (-7.3 cm^2/m^2, *p* <0.001) and a higher proportion were determined to be sarcopenic by the L3-SMI (*p* <0.001) (Table S2).

Association of historical decompensation events and Child-Pugh group

To assess the potential impact of historical clinical severity on baseline muscle health, we analysed the distribution of prior decompensation events across Child-Pugh classes (Table S3). Historical decompensation events (such as ascites, variceal bleeding, and hepatic encephalopathy) were equally distributed between patients currently classified as Child-Pugh A and B (*p* >0.05). The only exception was a single case of spontaneous bacterial peritonitis occurring in the Child-Pugh B group. Furthermore, there was no difference in the proportion of patients with Child-Pugh B status between the adverse MC and non-adverse MC groups (Table 2).

Discussion

In this prospective study, 150 patients with liver cirrhosis underwent MRI-based HCC surveillance concurrently with a concise MR protocol to assess body composition and muscle health. Although the majority of these patients were defined as having compensated cirrhosis, one in five (21%) presented with adverse MC, that is, a combination of high muscle fat and low volume defined using cut-points from the UK Biobank. In addition, more than half of the patients (54%) had low muscle volumes and a quarter (27%) had elevated muscle fat. Physical

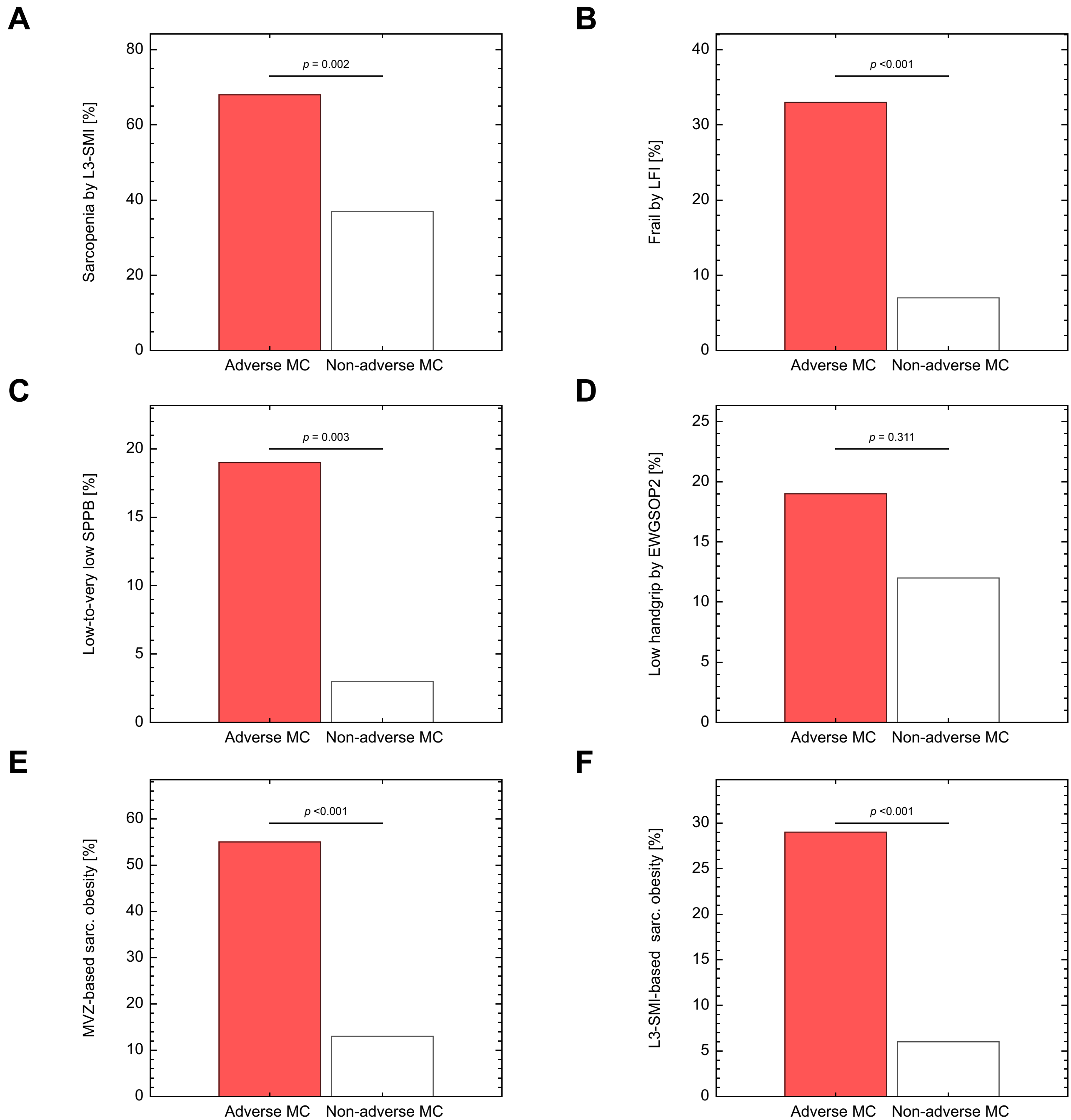

Fig. 4. The prevalence of subjects reaching frailty and functional test cut-points for the adverse muscle composition (MC) and non-adverse MC phenotypes. (A) Sarcopenia defined by the L3-SMI (X^2-test), (B) frail defined by the LFI (X^2-test), (C) low-to-very low physical function defined by the SPPB (X^2-test), (D) low handgrip defined by EWGSOP2 (X^2-test), (E) sarcopenic obesity by low muscle volume z-score and BMI ≥30 kg/m^2 (X^2-test), and (F) sarcopenic obesity by sarcopenia by L3-SMI and BMI ≥30 kg/m^2 (X^2-test). EWGSOP, European Working Group on Sarcopenia in Older People; L3-SMI, Skeletal Muscle Index at the third lumbar vertebra; LFI, Liver Frailty Index; SPBB, Short Physical Performance Battery.

frailty and functional tests revealed that patients with adverse MC exhibited higher frailty and poorer physical performance compared with those without adverse MC. Furthermore, sarcopenic obesity was more prevalent in patients with adverse MC. This difference was not found when comparing low muscle volume *vs*. normal muscle volume, suggesting that elevation of muscle fat is the primary driver of functional impairment. Notably, there was no difference in disease state, including Child-Pugh class, MELD score, previous decompensation, T2D, and BMI between the adverse MC and non-adverse MC groups, highlighting the unique information provided by the muscle phenotype. In addition, those with adverse MC also exhibited higher visceral adiposity, which is linked to a higher risk of cardiovascular events.[29]

Previous research has demonstrated that adverse MC is significantly associated with all-cause mortality in patients with

steatotic liver disease and in the general population, as well as poorer physical performance and increased metabolic comorbidities.[8–10] It remains to be assessed whether this holds true in patients with liver cirrhosis, or if cirrhosis-specific cut-points for adverse MC are required. A pilot study on patients with decompensated liver cirrhosis as well as emerging data on advanced fibrosis encourages us to continue our research in this area.[24,30] While this study reports baseline data, the ACCESS-ESLD design includes both a two-year follow-up by MRI and an additional 10-year follow-up by consulting patient records, allowing future exploration of the links to hard outcomes and of the validity of the MC phenotypes and their definitions in this patient group.[15] This is the first study to report on the prevalence of adverse MC in patients with compensated liver cirrhosis enrolled in HCC surveillance programmes. In contrast to the significant prevalence of adverse MC reported in the present study (21%; 64 ± 10 years), earlier studies on patients with similar age ranges (Fig. 2B) reported a 14% prevalence in patients with MASLD (64 ± 7 years) without liver cirrhosis,[10] and a 16% prevalence in patients with T2D (of which 59% were found to also have MASLD) in primary care (64 ± 8 years).[31] Moreover, recent pilot studies have reported prevalences of 39% (58 ± 9 years) and 41% (59 ± 5 years) in liver transplant candidates.[11,24] For comparison, 11% out of 40,000 subjects in the UK Biobank imaging substudy (64 ± 8 years) had adverse MC.[8] In a meta-analysis, the prevalence of sarcopenia in patients with liver cirrhosis was 37.5%; this included patients with more severe disease listed for liver transplantation and was mostly based on L3-SMI or similar measurements.[3]

In the present cohort, isolated low muscle volume (33%) was more prevalent than isolated high MFI (5%), while 21% of patients exhibited both. In the subgroup of patients with Child-Pugh B, 47% had high MFI. Contrasting with L3-based studies where myosteatosis (muscle fat) is reported to be more prevalent than sarcopenia.[12,32] However, comparisons should be made with caution as differences may stem from anatomical, methodological, and disease severity factors. Most existing studies utilise single-slice CT assessments on abdominal and paraspinal muscles at the L3 level. In contrast, our study uses volumetric MRI of the thigh. To investigate this discrepancy, we conducted a secondary analysis of MFI at the L3-level. While we found a very strong linear correlation to thigh MFI, the L3-MFI levels were systematically higher (mean +6.6 pp) and increased more rapidly (slope of 1.3 pp). This linear association is substantiated by data from a pilot study of patients listed for liver transplantation, where the average thigh MFI was 7.8%, and the average L3-MFI was 16.8% (matching the model prediction of 16.7%).[11] These findings indicate differences in fatty infiltration patterns across muscle groups. Based on these results, we hypothesise that the postural muscles of the trunk may accumulate fat earlier or more extensively than the locomotor muscles of the thighs, although this requires further dedicated study. Lipotoxicity, may play a role in the development of myosteatosis, and it has been suggested that different muscle groups exhibit varying patterns of vulnerability to such metabolic stress,[33] which could account for the variation observed here. In addition, the prevalence of myosteatosis is highly dependent on the threshold applied. To our knowledge, no validated MFI percentage threshold exists for the L3 level. Therefore, identifying myosteatosis at the L3 level may capture a different patient group than a thigh-based assessment. Our data show that thigh MFI is associated with functional measurements (LFI, SPBB, and gait speed), mirroring findings at the L3 level where muscle quality was more strongly linked to frailty than muscle mass.[12] Finally, our cohort consists of patients in HCC surveillance, 90% Child-Pugh Class A with a mean MELD 3.0 of 8.9, predominantly MASLD and ALD. This contrasts with studies featuring higher proportions of HCV and MELD and Child-Pugh scores.[12,32] This group of less severely ill patients may exhibit differences in muscle fat patterns. Future longitudinal studies are needed to determine whether myosteatosis at the thigh or L3 level better predicts long-term clinical outcomes and how these changes coincide throughout the natural history of the liver disease.

The MC phenotypes are based on the MVZ, a BMI- and sex-invariant muscle metric derived from the FFMV of the thighs, and MFI. These MR-based measurements were found to correlate with commonly used functional tests and panels, including the L3-SMI, HGS, LFI, and SPPB. The LFI and SPPB functional tests were most strongly correlated with the thigh MFI. HGS, the strength test, was most strongly correlated with the FFMV, consistent with previous research.[7,11] The single-slice abdominal muscle measurement, the L3-SMI, was most strongly correlated with the FFMV, followed by the MVZ and MFI. The stronger correlation between thigh FFMV and HGS and L3-SMI compared to the MVZ was expected, as these two measurements provide different physiological information. This correlation analysis underscores the added value of measuring muscle fat in addition to muscle volume, as muscle fat most strongly links to muscle function.

Sarcopenia is characterised by the progressive loss of muscle volume and function, and it is a well-established major complication of liver cirrhosis. Clinical practice guidelines suggest that objective measures of muscle content should be considered for assessing the risk of poor outcomes in patients with liver cirrhosis.[4] Currently, *ad hoc* analysis of single abdominal CT images is common, but CT is not recommended solely for assessing sarcopenia due to ionising radiation exposure.[4] These analyses typically rely on a small set of axial slices as proxies for the entire volume.[6] The most commonly used single-slice assessment of muscle loss in liver cirrhosis is the L3-SMI, which was also measured in the present study. Patients in the adverse MC group had a significantly lower L3-SMI, and within the adverse MC group there was a higher proportion of patients designated as sarcopenic using established sex-specific cut-points for the L3-SMI. Additionally, both FFMV and its corresponding z-score, MVZ, were strongly correlated with the L3-SMI, indicating that muscle content loss by the L3-SMI would probably also be identified using these thigh muscle volume measurements. Changes in muscle content identified through these thigh muscle measurements are probably more precise than those identified using the L3-SMI, as they rely on the whole thigh rather than a single abdominal slice.[6]

Current practice guidelines recommend US for HCC surveillance, but its effectiveness is compromised by hepatic fat and abdominal adipose tissue, which are expected to increase with the obesity epidemic. In this study, we explored an abbreviated MRI protocol for HCC surveillance. Although this analysis is not presented in this paper, others have investigated its merits.[14,34] and it is increasingly used in clinical

settings, especially when US is contraindicated or infeasible because of excess fat deposits. By integrating abbreviated MR-based HCC surveillance with a concise MR protocol for muscle health assessment, it is possible to effectively screen for major complications of liver cirrhosis, potentially enabling more comprehensive and effective approaches to patient care. The MR image processing time and operational costs were not formally assessed in this study. The cost-effectiveness and clinical value of these muscle heath assessments remain to be established in future evaluations.

The components of adverse MC, low muscle volume and high muscle fat, are increasingly recognised as modifiable therapeutic targets. This is particularly relevant for patients with liver cirrhosis, where a recent phase II trial using the oral androgen receptor agonist LPCN 1148 was the first to demonstrate simultaneous improvements in both muscle mass and fat infiltration.[35] This potential also extends also to metabolic diseases; for instance, tirzepatide treatment in patients with T2D was shown to improve MC.[36] Together, these studies highlight that the specific features defining adverse MC are treatable.

An interesting finding in our cohort was the high prevalence of historical decompensation events among patients currently classified as Child-Pugh A. We observed no significant difference in the history of events such as ascites, variceal bleeding, or hepatic encephalopathy between patients with Child-Pugh A and B at baseline (Table S3). This observation aligns with the Baveno VII framework of 'recompensation', which recognises that patients may transition from a decompensated state back to a compensated Child-Pugh A status following the removal of a primary insult, such as alcohol cessation or lifestyle changes.[37,38] Our analysis showed that the proportion of patients with Child-Pugh B status did not differ between the adverse and non-adverse MC groups (Table 2). This suggests that while liver function may improve over time (recompensation), the impact of prior clinical severity on muscle health probably persists or follows a different recovery trajectory. Therefore, MRI-based muscle phenotypes in this population represent a distinct marker of muscle health that is not merely a proxy for current Child-Pugh score or historical decompensation.

This study was designed to assess the prevalence and functional impact of adverse MC as a marker of sarcopenia across the general population of patients with liver cirrhosis, rather than focusing on specific aetiologies. We acknowledge that our cohort consists primarily of patients with Child-Pugh A cirrhosis and a high prevalence of overweight and obesity; consequently, further studies are needed in decompensated and liver transplant populations. Our results are representative of patients undergoing HCC surveillance in Sweden, reflecting a typical regional distribution of aetiologies where MASLD and ALD are dominant. While this provides a representative view of Northern European clinical practice, we recognise that these findings may not be directly extrapolatable to regions where the primary driver of cirrhosis differ, such as areas where viral hepatitis predominates.[39]

Furthermore, while a granular comparison between MASLD, MetALD, and ALD would be of interest, this was not possible because of a lack of reliable data on alcohol consumption for all patients at the time of initial diagnosis. However, recent evidence suggests that steatotic liver disease classifications are highly dynamic, with a significant proportion of patients shifting between MASLD, MetALD, and ALD categories over time.[37] Therefore, reporting aetiologies as defined in clinical practice at the time of inclusion is a pragmatic approach, and the inability to isolate a MetALD subgroup does not impact our primary conclusions regarding the broader cirrhosis population.

Additionally, the study was not powered to detect aetiology-specific variations in MC for rare conditions; thus, we cannot rule out the influence of specific underlying diseases on muscle phenotypes. Future multi-centre studies or pooled retrospective analyses will be required to provide the granular statistical power necessary for such subgroup evaluations. Finally, the cross-sectional nature of this study precludes the assessment of longitudinal muscle changes or clinical outcomes. However, as described in the study design, the ACCESS-ESLD study includes planned long-term follow-ups that will specifically address the prognostic significance of the baseline muscle phenotypes reported here.

In this prospective multi-centre study of patients with liver cirrhosis undergoing HCC surveillance by abbreviated MRI, it was demonstrated that by adding a concise MR protocol for quantifying body composition, 21% of the patients were found to have an adverse MC (defined as high muscle fat content and low MVZ with cut-points from the UK Biobank). Moreover, this subgroup exhibited higher frailty, poorer physical performance, a high proportion of sarcopenic obesity as well as visceral adiposity, none of which was related to the liver disease status as measured by routine clinical tests. A main conclusion of this work is that MC assessment can potentially be used to identify patients who may benefit from therapeutic interventions.

Affiliations

[1]*Department of Health, Medicine and Caring Sciences, Linköping University, Linköping, Sweden;* [2]*AMRA Medical AB, Linköping, Sweden;* [3]*Center for Medical Image Science and Visualization (CMIV), Linköping University, Linköping, Sweden;* [4]*Department of Medicine, Höglandssjukhuset Eksjö, Region Jönköping County Council, Eksjö, Sweden;* [5]*Department of Internal Medicine, Ryhov Hospital Jönköping, Region Jönköping County Council, Jönköping, Sweden;* [6]*Clinical Department of Gastroenterology and Hepatology in Linköping, Region Östergötland, Linköping, Sweden;* [7]*Wallenberg Center for Molecular Medicine (WCMM), Linköping University, Linköping, Sweden;* [8]*Institute of Medicine, Sahlgrenska Academy, University of Gothenburg, Göteborg, Sweden;* [9]*Department of Medicine, Sahlgrenska University Hospital, Göteborg, Sweden;* [10]*Clinical Department of Radiology in Linköping, Region Östergötland, Linköping, Sweden;* [11]*Clinical Department of Medical Radiation Physics, Region Östergötland, Linköping, Sweden;* [12]*Futurum, Academy for Healthcare, Region Jönköping County, Jönköping, Sweden;* [13]*The Jönköping Academy for Improvement of Health and Welfare, Hälsohögskolan, Jönköping University, Jönköping, Sweden*

Abbreviations

AAT, alpha-1 antitrypsin; ALD, alcohol-related liver disease; aMRI, abbreviated MRI; ANT, Animal Naming Test; ASAT, abdominal subcutaneous adipose tissue volume; CT, computed tomography; EWGSOP2, revised European working group in Sarcopenia in older people; FFMV, fat-free muscle volume; HCC, hepatocellular carcinoma; HGS, hand grip strength; L3-SMI, Skeletal Muscle Index at the third lumbar vertebra; LFI, Liver Frailty Index; MASLD, metabolic dysfunction-associated steatotic liver disease; MAsS, Muscle Assessment Score; MC, muscle composition; MELD, model for end-stage liver disease; MetALD, MASLD and increased alcohol intake; MFI, muscle fat infiltration; MRI,

magnetic resonance imaging; MVZ, muscle volume z-score; PDFF, proton density fat-fraction; QC, quality control; SPPB, Short Physical Performance Battery; T2D, type 2 diabetes; TIPS, transjugular intrahepatic portal shunt; US, ultrasonography; VAT, visceral adipose tissue volume.

Financial support

This study is supported by the Medical Research Council of Southeast Sweden, ALF Grants, County Council of Östergötland (Region Östergötland), The Swedish Medical Society, The Swedish Cancer Society (Cancerfonden), The Bengt Ihre Foundation, The Ruth and Richard Julin Foundation, The Swedish Research Council (Vetenskapsrådet/NT), and The Wallenberg Centre for Molecular Medicine, Linköping University.

Conflicts of interest

MMF is a shareholder and employee of AMRA Medical AB. PN has served on an advisory board for Boehringer Ingelheim. AC has received lecture fees from Norgine Denmark A/S. PL is shareholder of AMRA Medical AB. ODL is an employee, shareholder, and board member of AMRA Medical AB. ME has received lecture fees from Mediplast and Novo Nordisk and has served on an advisory board for Ipsen. The remaining authors declare no conflicts of interest.

Please refer to the accompanying ICMJE disclosure forms for further details.

Authors' contributions

Study concept and design: MFF, PN, PL, ND, SK, ODL, ME. Acquisition of data: all authors. Analysis and interpretation of data: MFF, PN, ODL, ME. Drafting of the manuscript: MFF, PN, ODL, ME. Critical revision of the manuscript for important intellectual content: all authors. Statistical analysis: MFF, ODL. Obtained funding: MFF, HS, PN, PL, MR, ND, SK, ODL, ME. Study supervision: MFF, ODL, ME. Administrative, technical, or material support: all authors. Approval of final draft: all authors.

Data availability

The data that support the findings of this study are available on reasonable request to the corresponding author (MF) or to the principal investigator (ME). The data are not publicly available owing to privacy or ethical restrictions.

Acknowledgements

We extend our gratitude to all research nurses involved, particularly Carola Fagerström, Camilla Holmgren, Agneta Aasa, and Sandra Bredén. We also acknowledge the important work of Forum Östergötland and the Linköping Biobank Facility.

Declaration of generative AI and AI-assisted technologies in the manuscript preparation process

During the revision of this work, the authors used Gemini (Google) in some sections to assist in improving linguistic clarity. After using this tool/service, the authors reviewed and edited the content as needed and take full responsibility for the content of the published article.

Supplementary data

Supplementary data to this article can be found online at https://doi.org/10.1016/j.jhepr.2026.102031.

References

Author names in bold designate shared co-first authorship

[1] James S, Abate D, Abate K, et al. Global, regional, and national incidence, prevalence, and years lived with disability for 354 diseases and injuries for 195 countries and territories, 1990–2017: a systematic analysis for the Global Burden of Disease Study 2017. Lancet 2018;392:1789–1858.

[2] Gines P, Krag A, Abraldes JG, et al. Liver cirrhosis. Lancet 2021;398:1359–1376.

[3] **Tantai X**, **Liu Y**, Yeo YH, et al. Effect of sarcopenia on survival in patients with cirrhosis: a meta-analysis. J Hepatol 2022;76:588–599.

[4] Lai JC, Tandon P, Bernal W, et al. Malnutrition, frailty, and sarcopenia in patients with cirrhosis: 2021 Practice Guidance by the American Association for the Study of Liver Diseases. Hepatology 2021;74:1611–1644.

[5] European Association for the Study of the Liver. EASL Clinical Practice Guidelines on the management of hepatic encephalopathy. J Hepatol 2022;77:807–824.

[6] Borga M, West J, Bell JD, et al. Advanced body composition assessment: from body mass index to body composition profiling. J Investig Med 2018;66:1–9.

[7] Linge J, Heymsfield SB, Dahlqvist Leinhard O. On the definition of sarcopenia in the presence of aging and obesity-initial results from UK Biobank. J Gerontol A Biol Sci Med Sci 2020;75:1309–1316.

[8] Linge J, Petersson M, Forsgren MF, et al. Adverse muscle composition predicts all-cause mortality in the UK Biobank imaging study. J Cachexia Sarcopenia Muscle 2021;12:1513–1526.

[9] Linge J, Nasr P, Sanyal AJ, et al. Adverse muscle composition is a significant risk factor for all-cause mortality in NAFLD. JHEP Rep 2023;5:100663.

[10] Linge J, Ekstedt M, Dahlqvist Leinhard O. Adverse muscle composition is linked to poor functional performance and metabolic comorbidities in NAFLD. JHEP Rep 2021;3:100197.

[11] Thuluvath AJ, Forsgren MF, Ladner DP, et al. Utilizing a novel MRI technique to identify adverse muscle composition in end-stage liver disease: A pilot study. Ann Hepatol 2024;29:101508.

[12] Di Cola S, D'Amico G, Caraceni P, et al. Myosteatosis is closely associated with sarcopenia and significantly worse outcomes in patients with cirrhosis. J Hepatol 2024;81:641–650.

[13] EASL. EASL Policy Statement: risk-based surveillance for hepatocellular carcinoma among patients with cirrhosis. 18 April 2023. https://easl.eu/publication/easl-policy-statement-risk-based/.

[14] Gupta P, Soundararajan R, Patel A, et al. Abbreviated MRI for hepatocellular carcinoma screening: a systematic review and meta-analysis. J Hepatol 2021;75:108–119.

[15] Nasr P, Forsgren M, Balkhed W, et al. A rapid, non-invasive, clinical surveillance for CachExia, sarcopenia, portal hypertension, and hepatocellular carcinoma in end-stage liver disease: the ACCESS-ESLD study protocol. BMC Gastroenterol 2023;23:454.

[16] Regionala cancercentrum (RCC). Levercellscancer–Nationellt vårdprogram. 2024. Version 4.0. https://kunskapsbanken.cancercentrum.se/diagnoser/levercellscancer/vardprogram/.

[17] Rinella ME, Lazarus JV, Ratziu V, et al. A multisociety Delphi consensus statement on new fatty liver disease nomenclature. J Hepatol 2023;79:1542–1556.

[18] West J, Dahlqvist Leinhard O, Romu T, et al. Feasibility of MR-based body composition analysis in large scale population studies. PLoS One 2016;11:e0163332.

[19] Linge J, Borga M, West J, et al. Body composition profiling in the UK Biobank imaging study. Obesity (Silver Spring) 2018;26:1785–1795.

[20] Borga M, Ahlgren A, Romu T, et al. Reproducibility and repeatability of MRI-based body composition analysis. Magn Reson Med 2020;84:3146–3156.

[21] Fowler KJ, Venkatesh SK, Obuchowski N, et al. Repeatability of MRI biomarkers in nonalcoholic fatty liver disease: The NIMBLE Consortium. Radiology 2023;309:e231092.

[22] Carey EJ, Lai JC, Wang CW, et al. A multicenter study to define sarcopenia in patients with end-stage liver disease. Liver Transpl 2017;23:625–633.

[23] Bhati C, Kirkman D, Forsgren MF, et al. Liver transplant recipients have worse metabolic body phenotype compared with matched non-transplant controls. JGH Open 2024;8:e70024.

[24] Forsgren MF, Pine S, Harrington CR, et al. Body composition and muscle composition phenotypes in patients on waitlist and shortly after liver transplant - results from a pilot study. BMC Gastroenterol 2024;24:356.

[25] Cruz-Jentoft AJ, Bahat G, Bauer J, et al. Sarcopenia: revised European consensus on definition and diagnosis. Age Ageing 2019;48:16–31.

[26] Kardashian A, Ge J, McCulloch CE, et al. Identifying an optimal Liver Frailty Index cutoff to predict waitlist mortality in liver transplant candidates. Hepatology 2021;73:1132–1139.

[27] Guralnik JM, Simonsick EM, Ferrucci L, et al. A short physical performance battery assessing lower extremity function: association with self-reported disability and prediction of mortality and nursing home admission. J Gerontol 1994;49:M85–M94.

[28] Campagna F, Montagnese S, Ridola L, et al. The animal naming test: an easy tool for the assessment of hepatic encephalopathy. Hepatology 2017;66:198–208.

[29] Linge J, Cariou B, Neeland IJ, et al. Skewness in body fat distribution pattern links to specific cardiometabolic disease risk profiles. J Clin Endocrinol Metab 2024;109:783–791.

[30] Balkhed W, Forsgren M, Cederborg A, et al. Muscle composition predicts adverse outcomes in chronic liver disease: a 5-year follow-up study. EASL SLD Summit 23–25 September 2025, Estoril, Portugal 2025:135.

[31] Balkhed W, Bergram M, Iredahl F, et al. Evaluating the prevalence and severity of metabolic dysfunction-associated steatotic liver disease in patients with type 2 diabetes mellitus in primary care. J Intern Med 2025;298:173–187.
[32] Montano-Loza AJ, Angulo P, Meza-Junco J, et al. Sarcopenic obesity and myosteatosis are associated with higher mortality in patients with cirrhosis. J Cachexia Sarcopenia Muscle 2016;7:126–135.
[33] Carter CS, Justice JN, Thompson L. Lipotoxicity, aging, and muscle contractility: does fiber type matter? Geroscience 2019;41:297–308.
[34] Park HJ, Jang HY, Kim SY, et al. Non-enhanced magnetic resonance imaging as a surveillance tool for hepatocellular carcinoma: comparison with ultrasound. J Hepatol 2020;72:718–724.
[35] Bruno BJ, Weavil JC, Ogle J, et al. Oral LPCN 1148 improves sarcopenia and hepatic encephalopathy in male patients with cirrhosis: a randomized, placebo-controlled phase 2 trial. Hepatology 2025;81:1764–1775.
[36] Sattar N, Neeland IJ, Dahlqvist Leinhard O, et al. Tirzepatide and muscle composition changes in people with type 2 diabetes (SURPASS-3 MRI): a post-hoc analysis of a randomised, open-label, parallel-group, phase 3 trial. Lancet Diabetes Endocrinol 2025;13:482–493.
[37] Israelsen M, Thorhauge KH, Andersen P, et al. Steatotic liver disease classification is dynamic, affecting clinical trial eligibility and subclass-specific treatments. Clin Gastroenterol Hepatol 2025;23:2509–2518.
[38] de Franchis R, Bosch J, Garcia-Tsao G, et al. Baveno VII - Renewing consensus in portal hypertension. J Hepatol 2022;76:959–974.
[39] Huang DQ, Terrault NA, Tacke F, et al. Global epidemiology of cirrhosis - aetiology, trends and predictions. Nat Rev Gastroenterol Hepatol 2023;20:388–398.

Keywords: Liver cirrhosis; Frailty; Muscle composition; HCC surveillance; Magnetic resonance imaging; Muscle volume; Muscle fat; Body composition; Adverse muscle composition.

Received 17 February 2026; received in revised form 2 September 2026; accepted 5 September 2026; Available online 11 September 2026

Journal of Hepatology, Volume *8*

Supplemental information

Prevalence and significance of combined low muscle volume and high muscle fat in patients with Child-Pugh A-B cirrhosis

Mikael Fredrik Forsgren, Daniel Sjögren, Henrik Stjernman, Patrik Nasr, Wile Balkhed, Anna Cederborg, Markus Holmberg, Wolf Bartholomä, Cecilia Jönsson, Peter Lundberg, Martin Rejler, Nils Dahlström, Stergios Kechagias, Olof Dahlqvist Leinhard, and Mattias Ekstedt

Prevalence and significance of combined low muscle volume and high muscle fat in patients with Child-Pugh A-B cirrhosis

Mikael Fredrik Forsgren, Daniel Sjögren, Henrik Stjernman, Patrik Nasr, Wile Balkhed, Anna Cederborg, Markus Holmberg, Wolf Bartholomä, Cecilia Jönsson, Peter Lundberg, Martin Rejler, Nils Dahlström, Stergios Kechagias, Olof Dahlqvist Leinhard, Mattias Ekstedt

Table of contents

Supplementary tables... 2

Supplementary figures 6

Supplementary tables

Table S1 Complete list of ranked liver cirrhosis aetiologies in alphabetical order.

**Aetiologies**	**n**
AAT-deficiency	1
AIH	9
AIH-PBC overlap	1
AIH, ALD, MASLD	1
ALD	50
ALD, HBV, HCV	1
ALD, HCV	1
ALD, HFE	1
ALD, MASLD	9
ALD, MASLD, HCV	1
ALD, MASLD, HCV, HBV	1
Budd-Chiari secondary to polycythemia vera	1
Cryptogenic	7
CVID	1
HCV	4
HCV, ALD	5
HCV, ALD, MASLD	2
HFE	1
MASLD	36
MASLD, ALD	5
MASLD, Cryptogenic	1
MASLD, Other	1
Neonatal cholestasis	1
Other	1
PBC	5
PBC, AIH	1
Primary ciliopathy	1
SSC	1

AAT, alpha-1 antitrypsin; AIH, autoimmune hepatitis; ALD, alcohol-related liver disease; CVID, common variable immunodeficiency; HBV, hepatitis-B virus; HCV, hepatitis-C virus; HFE, hereditary hemochromatosis; MASLD, metabolic-dysfunction associated steatotic liver disease; PBC, primary biliary cholangitis; SSC, secondary sclerosing cholangitis.

Table S2 Patient demographics, body composition, and functional test differences between the low and normal muscle volume. Data presented as mean (standard deviation), n (%), or median [range] as applicable. 147 patients had a complete thigh muscle assessment.

	**Low Muscle Volume** n = 80 (54%)	**Normal Muscle Volume** n = 67 (46%)	**P-values**	**Test**
Sex [male]	50 (32%)	35 (52%)	0.210	Chi2
Age [years]	65.8 (8.8)	62.8 (12.3)	0.088	T-test
BMI [kg/m^2]	28.5 (5.5)	30.3 (5.6)	**0.046**	T-test
Disease history				
Diabetes Type 2	32 (37%)	25 (37%)	0.739	Chi2
Primary aetiology ALD	44 (55%)	19 (28%)	**0.001**	Chi2
Primary aetiology MASLD	15 (19%)	27 (40%)	**0.004**	Chi2
Primary aetiology Hepatitis C	6 (8%)	5 (7%)	0.993	Chi2
Primary aetiology AIH or PBC	8 (10%)	8 (12%)	0.707	Chi2
Primary other aetiology	7 (6%)	8 (12%)	0.525	Chi2
Decompensation event prior to baseline	26 (33%)	19 (28%)	0.587	Chi2
Child-Pugh Class B	9 (11%)	6 (9%)	0.691	Chi2
MELD 3.0 [-]	8 [7, 10]	8 [7, 9]	0.885	Mann-Whitney U
MRI-based body composition				
Visceral Adipose Tissue volume [L]	5.8 (2.7)	5.5 (2.6)	0.574	T-test
Abdominal Subcutaneous Adipose Tissue volume [L]	8.9 (3.9)	9.2 (4.6)	0.646	T-test
Total Thigh Fat-free Muscle volume [L]	9.3 (2.2)	11.2 (2.6)	**<.001**	T-test
Mean Anterior Thigh Muscle Fat Infiltration [%]	9.0 (3.0)	6.9 (2.0)	**<.001**	T-test
Liver Proton Density Fat Fraction [%]	3.7 [2.2, 7.3]	3.3 [2.1, 5.6]	0.448	Mann-Whitney U
L3 Skeletal Muscle Index (L3-SMI) [cm^2/m^2]	43.6 (9.5)	50.9 (10.0)	**<.001**	T-test
Muscle Assessment Score (MAsS)				
Muscle Volume Z Score [SDs]	-1.6 (0.9)	0.3 (0.7)	**<.001**	T-test

Mean Anterior Thigh Muscle Fat Infiltration (sex-adjusted) [pp]	1.1 (2.8)	-1.1 (1.8)	**<.001**	T-test
Physical and cognitive function tests				
Animal Naming Test (ANT)	23 [18, 28]	21 [19, 25]	0.696	T-test
Short Physical Performance Battery (SPPB)	10 [9, 12]	11 [9, 12]	0.405	Mann-Whitney U
Liver Frailty Index (LFI)	3.7 [3.3, 4.1]	3.5 [3.1, 4.0]	0.108	Mann-Whitney U
Hand grip strength (HGS)	34 [24, 39]	36 [25, 46]	0.114	Mann-Whitney U
Dichotomous physical and cognitive function outcomes				
Low ANT	7 (9%)	6 (9%)	0.920	Chi2
Sarcopenia by L3-SMI	49 (62%)	14 (21%)	**<0.001**	Chi2
Low to very low physical performance by SPPB	6 (8%)	2 (3%)	0.222	Chi2
Frail by LFI	11 (14%)	7 (11%)	0.530	Chi2
Low hand-grip strength by HGS	11 (14%)	9 (14%)	0.960	Chi2

ALD, alcohol-related liver disease; MASLD, metabolic-dysfunction associated steatotic liver disease; AIH, autoimmune hepatitis; PBC, primary biliary cholangitis; MELD, Model for End-stage Liver Disease; ANT, Animal Naming Test; SPPB, Short Physical Performance Battery; LFI, Liver Frailty Index; HGS, Hand Grip Strength; L3-SMI, Skeletal Muscle Index at the 3rd Lumbar vertebrae.

Table S3 History of decompensation events prior to inclusion against the Child-Pugh score at inclusion. Data presented as n (%), p-value for fraction of historical events in the Child-Pugh A versus Child-Pugh B group using the Chi^2-test.

**Decompensation history** (prior to inclusion)	**Total**	**A5**	**A6**	**B7**	**B8**	**B9**	**P-value** (Child-Pugh A vs. B)
Clinical ascites	39 (26%)	25 (64%)	7 (18%)	5 (13%)	2 (5%)	0	0.054
Acute kidney injury	17 (11%)	12 (71%)	4 (24%)	1 (6%)	0	0	0.548
Variceal bleeding	14 (9%)	6 (43%)	5 (36%)	3 (21%)	0	0	0.134
Encephalopathy	8 (5%)	3 (38%)	3 (38%)	1 (13%)	0	1 (13%)	0.146
TIPS treatment	4 (3%)	1 (25%)	3 (75%)	0	0	0	0.499
Portal vein thrombosis	3 (2%)	2 (67%)	1 (33%)	0	0	0	0.560
Spontaneous bacterial peritonitis	1 (1%)	0	0	0	1 (100%)	0	**0.003**
Varices	69 (46%)	50 (72%)	12 (17%)	6 (9%)	1 (1%)	0	0.956

Supplementary figures

Fig. S1 Correlation analysis of muscle measurements to functional tests.

Spearman rank correlation analysis between thigh Muscle Fat Infiltration (A, D, G), thigh Muscle Volume Z-score (B, E, H), and thigh Fat-Free Muscle Volume (C, F, I), against the L3 Skeletal Muscle Index (A, B, C), L3 Skeletal Muscle Fat Infiltration (D, E, F), and the Liver Frailty Index (G, H, I).

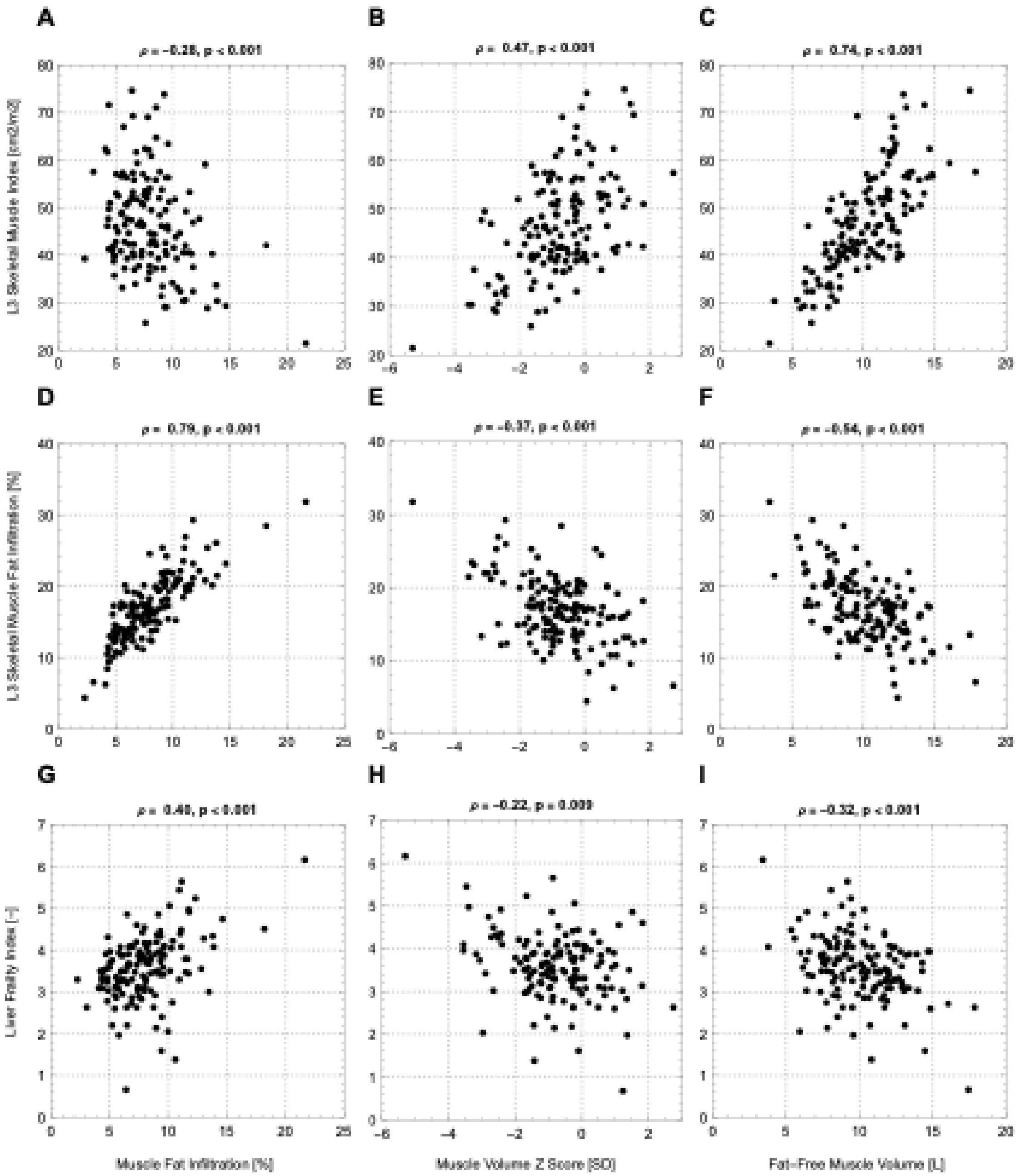

Fig. S2 Correlation analysis of muscle measurements to functional tests.

Spearman rank correlation analysis between thigh Muscle Fat Infiltration (A, D, G), thigh Muscle Volume Z-score (B, E, H), and thigh Fat-Free Muscle Volume (C, F, I), against the Gait Speed (A, B, C), Hand Grip Strength (D, E, F), and the Short Physical Performance Battery (G, H, I).

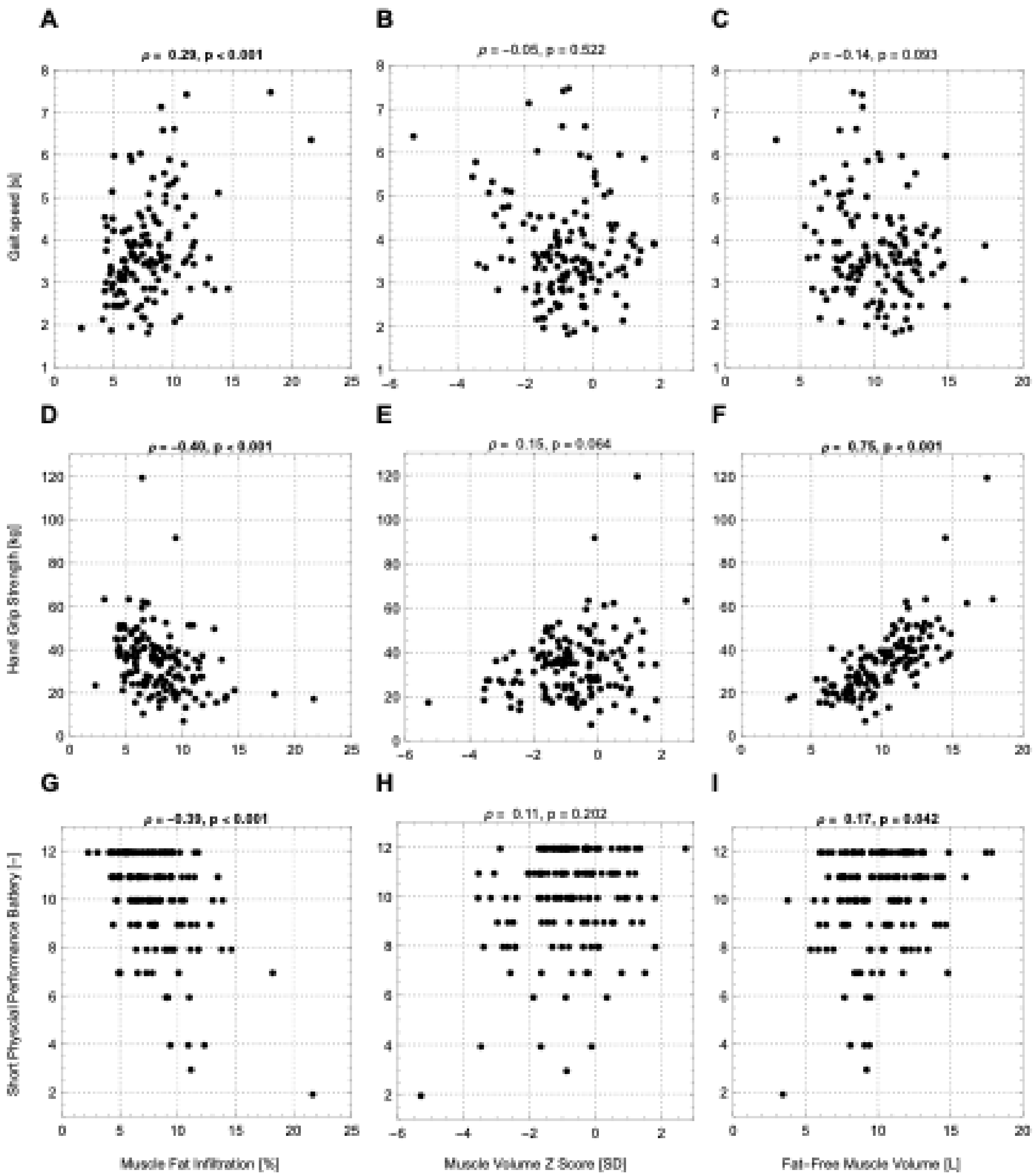

Supplement: Supplementary file 3 [file mmc4.pdf]
