# Supplementary material for: Prevalence and significance of combined low muscle volume and high muscle fat in patients with Child-Pugh A-B cirrhosis
Source: JHEP Rep. 2026 Sep 11;8(12):102031. doi: 10.1016/j.jhepr.2026.102031 (PMC13742102; doi:10.1016/j.jhepr.2026.102031)
Supplement: Supplementary file 4 [file mmc2.docx]

**JHEP Reports**

**CTAT methods**

Tables for a “Complete, Transparent, Accurate and Timely account” (CTAT) are now mandatory for all revised submissions. The aim is to enhance the reproducibility of methods.

- Only include the parts relevant to your study
- Refer to the CTAT in the main text as ‘Supplementary CTAT Table’
- Do not add subheadings
- Add as many rows as needed to include all information
- Only include one item per row

**If the CTAT form is not relevant to your study, please outline the reasons why:**

This study did not involve laboratory-based experimental assays, cell lines, animal models, or primary biological reagents. The methodology relies entirely on commercial and standardized data/image analysis. Therefore, the experimental sections are not applicable. To ensure transparency, in the "Software" section of this table we have listed the software used for image analysis as well as statical analysis (which are also stated in the manuscript).

1.1 **Antibodies**

| **Name** | **Citation** | **Supplier** | **Cat no.** | **Clone no.** |
| --- | --- | --- | --- | --- |
| N/A |  |  |  |  |

1.2 **Cell lines**

| **Name** | **Citation** | **Supplier** | **Cat no.** | **Passage no.** | **Authentication test method** |
| --- | --- | --- | --- | --- | --- |
| N/A |  |  |  |  |  |

1.3 **Organisms**

| **Name** | **Citation** | **Supplier** | **Strain** | **Sex** | **Age** | **Overall n number** |
| --- | --- | --- | --- | --- | --- | --- |
| N/A |  |  |  |  |  |  |

1.4 **Sequence based reagents**

| **Name** | **Sequence** | **Supplier** |
| --- | --- | --- |
| N/A |  |  |

1.5 **Biological samples**

| **Description** | **Source** | **Identifier** |
| --- | --- | --- |
| N/A |  |  |

1.6 **Deposited data**

| **Name of repository** | **Identifier** | **Link** |
| --- | --- | --- |
| N/A |  |  |

1.7 **Software**

| **Software name** | **Manufacturer** | **Version** |
| --- | --- | --- |
| AMRA Researcher | AMRA Medical AB, Linköping, Sweden | 3 |
| Mathematica | Wolfram Research Inc, Champaign, IL, USA | 12.2 |

1.8 **Other (*e.g*. drugs, proteins, vectors etc.)**

| N/A |  |  |
| --- | --- | --- |
|  |  |  |

1.9 **Please provide the details of the corresponding methods author for the manuscript:**

Mikael Forsgren, Department of Health, Medicine and Caring Sciences, Linköping University, SE-581 83 Linköping, Sweden

**2.0	Please confirm for randomised controlled trials all versions of the clinical protocol are included in the submission. These will be published online as supplementary information.**

N/A
